# Life With Corona: Increased Gender Differences in Aggression and Depression Symptoms Due to the COVID-19 Pandemic Burden in Germany

**DOI:** 10.3389/fpsyg.2021.689396

**Published:** 2021-07-27

**Authors:** Liliana Abreu, Anke Koebach, Oscar Díaz, Samuel Carleial, Anke Hoeffler, Wolfgang Stojetz, Hanna Freudenreich, Patricia Justino, Tilman Brück

**Affiliations:** ^1^Development Research Group, Department of Politics and Public Administration, University of Konstanz, Konstanz, Germany; ^2^Clinical Neuropsychology, Department of Psychology, University of Konstanz, Konstanz, Germany; ^3^ISDC – International Security and Development Center, Berlin, Germany; ^4^Leibniz Institute of Vegetable and Ornamental Crops, Großbeeren, Germany; ^5^World Institute for Development Economic Research, United Nations University, Helsinki, Finland; ^6^Natural Resources Institute, University of Greenwich, Chatham Maritime, United Kingdom

**Keywords:** aggression, anxiety, depression, somatization, mental health, COVID-19 pandemic, gender differences

## Abstract

Gender differences (GD) in mental health have come under renewed scrutiny during the COVID-19 pandemic. While rapidly emerging evidence indicates a deterioration of mental health in general, it remains unknown whether the pandemic will have an impact on GD in mental health. To this end, we investigate the association of the pandemic and its countermeasures affecting everyday life, labor, and households with changes in GD in aggression, anxiety, depression, and the somatic symptom burden. We analyze cross-sectional data from 10,979 individuals who live in Germany and who responded to the online survey “Life with Corona” between October 1, 2020 and February 28, 2021. We estimate interaction effects from generalized linear models. The analyses reveal no pre-existing GD in aggression but exposure to COVID-19 and COVID-19 countermeasures is associated with sharper increases in aggression in men than in women. GD in anxiety decreased among participants with children in the household (with men becoming more anxious). We also observe pre-existing and increasing GD with regards to the severity of depression, with women presenting a larger increase in symptoms during the hard lockdown or with increasing stringency. In contrast to anxiety, GD in depression increased among participants who lived without children (women > men), but decreased for individuals who lived with children; here, men converged to the levels of depression presented by women. Finally, GD in somatic symptoms decreased during the hard lockdown (but not with higher stringency), with men showing a sharper increase in symptoms, especially when they lived with children or alone. Taken together, the findings indicate an increase in GD in mental health as the pandemic unfolded in Germany, with rising female vulnerability to depression and increasing male aggression. The combination of these two trends further suggests a worrying mental health situation for singles and families. Our results have important policy implications for the German health system and public health policy. This public health challenge requires addressing the rising burden of pandemic-related mental health challenges and the distribution of this burden between women and men, within families and for individuals who live alone.

## Introduction

More than 1 year has passed since the beginning of the COVID-19 pandemic and evidence on its profound psychological impacts is emerging rapidly from around the world (Wang C. et al., 2020; Williams et al., 2020). So far, the life-threatening and traumatic nature of the pandemic, as well as the increased stress load imposed by measures to contain the SARS-CoV-2 virus, the coronavirus causing COVID-19 (hereafter referred to as “the coronavirus”), are causing a deterioration in mental health (Xiang et al., 2020). Recent studies document higher levels of stress and anxiety, loneliness and insomnia, somatization, and depressive symptoms, as well as symptoms of post-traumatic stress (Ko et al., 2020; Ran et al., 2020; Shelef and Zalsman, 2020). A meta-study (Wu et al., 2021) conducted in May 2020 evaluated over 10 studies on anxiety and depression since the onset of the COVID-19 pandemic and found prevalence rates of over 30%. Accordingly, recent meta-analyses estimated the prevalence of depression in the general population during the pandemic at 25% (95% confidence interval: 18–33%) (Bueno-Notivol et al., 2021) and 33.7% (95% confidence interval: 27.5–40.6) (Salari et al., 2020); pre-pandemic prevalence rates between 1994 and 2014 across 30 communities averaged at 12.9% (95% confidence interval: 11.1–15.1%) and data from Global Burden of Disease showed a proportion of 3.4% in 2017 (Ritchie and Roser, 2018).

Given the gender-specific challenges associated with the pandemic, the latter may also impact mental health differently by gender. Gender, beyond the biological definition of sex, refers to socially allocated roles, behaviors, identities, and expectations. In the literature, gendered behavior is understood as a cultural phenomenon rather than merely biological, given its highly relational nature (Flanagan, 2012). Gender differences (GD) in mental health outcomes have been established previously. Anxiety and mood disorders have been shown to be more prevalent among women than men (Rosenfield and Mouzon, 2013), while externalizing behavior or aggression and substance use disorders are more prevalent among men than women (Seedat et al., 2009; Boyd et al., 2015). To varying extents, these differences are assumed to be the result of sex-specific genetic (Kang et al., 2020), epigenetic (Hodes et al., 2017), neural (Stewart et al., 2010), reproductive (Li and Graham, 2017), and social factors, e.g., social roles and gender norms (Alon et al., 2020). It is widely acknowledged that gender is experienced not just individually but also socially. These social roles were found to partially explain GD in the perception of psychological distress (Simon, 1995), and GD in mental health is one of the facets where they manifest themselves. As the pandemic imposes different stressors on individuals with different (gendered) roles due to, for example, unemployment, home schooling, and working from home, differences in the impact of psychological distress on mental health may emerge. However, to date, we have limited evidence about the pandemic-related stressors that affect gender roles differentially, and how they impact GD in mental health.

In summary, the aim of this paper is to address knowledge gaps about GDs of mental health outcomes that emerged with the COVID-19 pandemic. Specifically, we analyze GD in aggression, anxiety, and depression symptoms, as well as the somatic symptom burden for adult men and women in Germany during the winter period of 2020/2021. For the purpose of the current study, we categorize gender as male, female or other and analyze data on the binary spectrum. This is undoubtedly a coarse categorization, since growing empirical evidence affirms that gender is a non-binary construct. Recent awareness of gender diversity draws attention to the experience and rights of transgenders and individuals who perceive their gender identity as neither entirely male nor female (Ainsworth, 2015; Cameron and Stinson, 2019). In the methods and limitations sections, we describe why we chose the binary construct to answer our research questions, although we agree that research should move toward more gender inclusivity. To examine the impact of the COVID-19 pandemic, we specify three sets of analyses: first, the nature and intensity of containment measures imposed in Germany, comparing outcomes during *light* vs. *hard lockdowns* and across stringency levels of measures to contain the virus using the Oxford Stringency Index; second, exposure to the coronavirus by testing positive for the virus, knowing someone who died due to the pandemic and suffering income losses during the pandemic; third, household characteristics–being the main provider, living vs. not living with children, and living alone vs. with others in interaction with the stringency index.

### GD in Aggression

Aggression can be defined as any behavior intended to cause harm in others motivated either reactively, thus occurring as a response to a perceived threat, or instrumentally/proactively (Anderson and Bushman, 2002). Increasing evidence points to the rewarding and thus self-perpetuating nature of aggression (Nell, 2006; Elbert et al., 2010, 2018; Koebach and Elbert, 2015; Golden and Shaham, 2018; Golden et al., 2019; Koebach et al., 2021). In recent aggression models, aggressive behavior is described as a consequence of situational (e.g., stress, frustration, discomfort, threatening stimuli) and personal factors (e.g., traits, attitudes, gender, trauma history), as well as internal states (e.g., cognition, affect, and arousal; e.g., Anderson and Bushman, 2002; Bushman, 2016; Elbert et al., 2018). In contrast, anger is considered a social emotion manifesting as a state or trait (Spielberger et al., 1983, 1995), and predisposing for an aggressive action in response to a (perceived) threat (Bettencourt et al., 2006). Inherent to the survival mode (Chemtob et al., 1997; Novaco and Chemtob, 1998) and with its property to suppress fear (Foa et al., 1995; Feeny et al., 2000), it is highly prevalent in trauma-exposed individuals (for review see Orth and Wieland, 2006). Within this framework, aversive situations such as the COVID-19 pandemic and its circumstances, e.g., confinement or economic hardship, may impose an increased level of threat, frustration and discomfort. We theorize that COVID-19-related stressors stimulate cognitive, emotional, and physiological reactions that are associated with threat, and thus trigger fight-or-flight tendencies that lead to a higher level of anger and reactive physical aggression (Gelles, 1993; Allen et al., 2018; Elbert et al., 2018).

In line with this hypothesis, Ye et al. (2021) found an increase of online aggressive behavior associated with fear about contagion with COVID-19. In 2016, during an epidemic of the Middle East Respiratory Syndrome (MERS), Jeong et al. (2016) examined the effects of a 2-week isolation period on anger and anxiety, and found about 16% of participants experienced anger when isolated due to MERS virus exposure. Several health experts and scientists have also observed increasing rates of family violence during the COVID-19 pandemic, particularly in situations of more stringent quarantines (Fraser, 2020; Perez-Vincent et al., 2020; Telles et al., 2020; Ebert and Steinert, 2021). In a recent meta-analysis, Piquero et al. (2021) found strong evidence for a moderate increase in domestic violence as a result of the pandemic based on 18 studies from the United States (*n* = 12), Mexico (*n* = 1), Argentina (*n* = 1), India (*n* = 1), Australia (*n* = 1), and Europe (*n* = 2). A study in Germany estimated the prevalence of violence against women and children during the pandemic, reporting 3.1% of women suffered verbal and physical conflict during the previous month; 7.8% reported emotional abuse; 3.1% felt threatened by their partner; and 6.7% reported child corporal punishment (Ebert and Steinert, 2021). The authors concluded that the risk of violence was more than double in households in quarantine, compared with households not in quarantine. Accordingly, Leslie and Wilson (2020) reported an increase of 7.5% of police calls in the United States due to an incident of intimate partner violence (IPV) during the first months of the pandemic. With regard to crime rates, evidence shows an overall decline in almost all types of crime during lockdowns, with exception to homicides and cyber crime (Halford et al., 2020; Hodgkinson and Andresen, 2020; Buil-Gil et al., 2021; Scott and Gross, 2021; Sutherland et al., 2021). A study from Australia recently showed that this effect might reverse once the measures are lifted (Andresen and Hodgkinson, 2020), but longer-term developments in crime indices remain to be explored. Thus, the literature suggests a shift of violence from the streets into the homes.

Traditionally, boys and men have been considered more aggressive than girls and women (Leslie and Wilson, 2020). However, more recent approaches claim gender-specific types of aggression with men being directly aggressive and women indirectly (e.g., spreading rumors) have come to the fore (Lagerspetz et al., 1988). This is also reflected in how women respond to anger: while it is recognized that men and women generally display comparable levels of anger (Deffenbacher et al., 1996), men tend to externalize their aggressive feelings more (Archer, 2004; Björkqvist, 2018), while women may respond to provocation with more anxiety and fear (Björkqvist, 2018). In a large sample from Denmark (>10,000 participants), found that hospitalization due to interpersonal violence predicted criminal behavior in men and self-harm in women. Moreover, about 80% of all global homicides are perpetrated by men (Global Study on Homicide, 2019). Yet, there are conditions when GD in aggression disappear. In a meta-analysis, Knight et al. (2002) found that GD in aggression was most pronounced when the context allowed for variance in emotional arousal (rather than secure or highly arousing situations). This is also in line with findings from war-affected men and women who both presented similar levels of appetitive aggression after involvement to similar levels of trauma and violent fighting (Augsburger et al., 2015; Meyer-Parlapanis et al., 2016). GD in aggression have been argued to be associated with biological (e.g., Turanovic et al., 2017; Denson et al., 2018; Ling et al., 2019), psychological (e.g., as sequelae of trauma) and social factors (e.g., social learning, etc.). Further, researchers plausibly theorize that GD in aggression are the result of sexual selection throughout evolution (Archer and Webb, 2006; Elbert et al., 2018). Indirect/female forms of aggression are psychologically not less harmful (see Eisenberger and Lieberman, 2004; Eisenberger, 2012; Norman et al., 2012; Arseneault, 2017; Começanha et al., 2017; on social pain), but physical aggression and crime on average present higher societal costs and escalate more often into extreme forms requiring hospital admission, psychological treatment, restorative justice, isolation of perpetrators/imprisonment, etc. (Forum on Global Violence Prevention, 2011). Building on these findings, we focus in this paper on GD in anger and physical aggression, and postulate that, in Germany, men may respond with more anger and physical aggression to the stress caused by the pandemic.

### GD in Anxiety

Symptoms of Generalized Anxiety Disorder (GAD) include restlessness, fatigue, excessive anxiety and worry, impaired concentration, and difficulty sleeping. About 5–6% of the population is estimated to present the full clinical diagnosis of GAD (Kessler et al., 1994). The COVID-19 pandemic has increased fear of acute threat of infection and death, which has been magnified by secondary stressors, e.g., social distancing, lockdowns, economic insecurity and unemployment. As a result, the incidence rates of anxiety have increased since the start of the pandemic (Alonzi et al., 2020; Bäuerle et al., 2020a; Canet-Juric et al., 2020; Kazmi, 2020; Mazza et al., 2020; Olaseni, 2020; Ozamiz-Etxebarria, 2020; Ausín et al., 2021; Moore et al., 2021; Msherghi et al., 2021).

Furthermore, anxiety, GAD in particular, is about 2–3 times more prevalent in women than in men (Beesdo et al., 2010; McLean et al., 2011); for review see Jalnapurkar et al. (2018). Differential biological, psychological, and social functioning have been found to underlie GD in anxiety. A large body of evidence emphasizes specific effects of reproductive hormones (Altemus, 2006; Altemus et al., 2014), e.g., estrogen that modulates brain regions relevant to the extinction of fear (Garcia et al., 2018), or testosterone which has anxiolytic effects (McHenry et al., 2014). Differential vulnerability to trauma and exposure to everyday stressors may further increase GD in anxiety (Donner and Lowry, 2013; Durbano, 2015). Gender theories emphasizes the identification of sex roles as factors determining GD in anxiety (Altemus, 2006; Altemus et al., 2014), namely when discussing how etiological factors of anxiety and individual differences are moderated by socialization processes (social, cultural, and developmental) and gender-specific expectations (McLean and Anderson, 2009). Traditionally, gender role theory advocates that, in socialization processes, men and women are socially prescribed with certain behaviors, traits, and skills, with considerable evidence reporting that gender roles significantly influence symptoms of anxiety (Bem, 1981). For instance, expression of anxiety is inconsistent with male gender roles, and anxiety may therefore be less tolerated in men (Chambless and Mason, 1986; Ollendick et al., 2002). Indeed, the magnitude of GD depends on the type of anxiety (Bander and Betz, 1981; Moscovitch et al., 2005).

Since the beginning of the COVID-19 pandemic, several studies have discussed the increased vulnerability of women during the crisis, as women tend to work in jobs that require face-to-face interaction, depend on part-time employment, and manage both family and work (Olaseni, 2020; Sánchez-Teruel, 2021). Szabo et al. (2020) investigated stress levels in 1,552 Hungarians during the first month of the COVID-19 crisis and found that women were more worried than men (Szabo et al., 2020) about the consequences of the pandemic. Frederiksen and Gomez (2020) found that women tend to be worried more about someone in their family getting infected by COVID-19 and about income decrease (50% vs. 42%). Losada-Baltar et al. (2020) showed that feelings of loneliness and psychological distress were higher in women in Spain. Higher stress levels in women during the early stage of the pandemic were also found in China (Yan et al., 2021). However, these studies are not able to identify the effect of the pandemic on GD in anxiety. So far, only the study of Ausín et al. (2021) investigated gender-specific consequences in mental health due to the pandemic, in the period immediately after the declaration of the state of emergency in Spain. We extend this analysis to another setting (Germany), focus on longer-term effects, and consider various pandemic-related stressors.

### GD in Depression

Depression refers to symptoms like depressed mood, loss of interest and pleasure, negative feelings and thoughts, and problems with sleeping and concentration, amongst others. Clinically relevant levels require these symptoms to persist for at least half a day and more than half of the days in a given time frame. Major depression is amongst the most prevalent mental disorders and a complex biopsychosocial interaction underlies the development of symptoms. In the advent of experimental psychology, Seligman (1972) introduced the concept of learned helplessness as he found that dogs exposed to electric shocks in an inescapable situation would later fail to escape electric shocks even when escape was possible (Overmier and Seligman, 1967). In humans, it was found that the attributional style (internal, global, and stable) is critical to whether subjects are able to cope with stressful situations (Abramson et al., 1978; Alloy, 1982; Raps et al., 1982). This model presents a prominent environmental theory for depression and its treatment in behavioral therapy at present (Rubenstein et al., 2016), besides other approaches that focus on traumatic or chronic environmental stressors (McCullough, 2003; O’Leary and Cryan, 2013; Wiborg, 2013). As with the electric shocks, the pandemic has been imposed on individuals as a sequence of inescapable and unavoidable adverse events emerging in the form of the threat of infection, lockdowns, economic crisis, and restrictions to individual freedoms. The ability of individuals to cope with these stressors is subject to personal characteristics. Studies comparing the prevalence of depressive symptoms before and after the start of the pandemic suggest an increase in depression since the pandemic. This has been in the order of around 10.1% (Bretschneider et al., 2017) before the pandemic and 14.3% during the pandemic (Bäuerle et al., 2020b) in Germany. An accumulation of depression symptoms was also reported in the United States, with a threefold increase during COVID-19 pandemic (Ettman et al., 2020).

Concerning the GD of depression during the pandemic, two studies from China found that women showed higher prevalence of depression during the crisis (Wang C. et al., 2020; Zhang and Ma, 2020). In a large online survey from Italy (N > 18,000), Rossi et al. (2020) found women were more likely to display higher levels of depression. GD in depression represent a major health disparity, as women suffer about twice as frequently from major depression than men (Weissman and Klerman, 1977; Salk et al., 2017). Hammarström et al. (2009) conducted a literature review on explanatory models for GD in depression. The authors found that the majority of studies focused on a biomedical explanation (Hammarström et al., 2009), followed by sociocultural and psychological models that were superior on intersectionality and multifactoriality. Converging evidence of recent studies emphasize the interaction of environmental stress (e.g., childhood adversity, physical, sexual or emotional abuse, or neglect) and biological vulnerability (e.g., due to sex hormones, inflammation, etc.; for review see ref). Interestingly, studies consistently report narrowing GD in depression when accompanied by changes in traditional gender roles (Wickramaratne et al., 1989; Joyce et al., 1990; Seedat et al., 2009).

Given the higher depression rates in women before and during the pandemic, studies that emphasize GD in depression fail to reflect whether this is exaggerated due to the pandemic. Only Ausín et al. (2021) investigated the change in GD in depression due to the pandemic in Spain but did not find any evidence. To extend their findings, we investigate the differential impact of the pandemic, its countermeasures and related stressors, as well as household characteristics as moderators in Germany.

### GD in Somatization

Somatic symptoms are common in medical, psychiatric, and social conditions, and are associated with higher levels of stress, decreased quality of life, and an increased use of health structures (Kroenke et al., 1990, 1997, 2010; Simon et al., 1999; Barsky et al., 2005; Rief et al., 2005; Fink et al., 2007; Kohlmann et al., 2013). This outcome has been subject to limited research in gender studies and during the pandemic. However, the few studies carried out suggest that COVID-19 may have considerable and gendered effects on somatization. Women and men reportedly experience somatic symptoms differently. Women tend to report somatic symptoms more frequently than men and experience them more intensively (Barsky et al., 2001). The reasons presented for these differences vary widely across studies, since the same sensation may be differently described and labeled by women and men. Pennebaker and Roberts (1992) suggested that women use both situational information (external) and somatic (internal) signs to describe symptoms, while men rely more on internal signs. Again, gender roles may enforce GD in somatization. According to Ehlers (1993), women receive more positive reinforcement for expressing somatic symptoms than men, which may reinforce self-focus and partially explain GD in somatization, while men might suppress those symptoms more often, since they feel more discouraged from expressing them (Watt et al., 1998). Unlike mental health problems, somatic symptoms may not trigger stigmatization to the same extent, and we include them in this study as a global health indicator. Taking this into account, we seek to clarify GD in somatization associated with the COVID-19 pandemic and determine if these differences increased before and after the introduction of stricter measures to control the spread of the virus.

## Materials and Methods

### Setting

Similar to other countries, the German federal and state governments, as well as local authorities, responded to the COVID-19 crisis by imposing countermeasures that included closures of schools and non-essential services, travel restrictions, mandatory self-isolation for travelers, and prohibition of gatherings. The second round of the Life with Corona (LwC) survey (see below) was launched during what was referred to as a light lockdown in October 2020: restaurants and cafés could only sell takeaway food and a maximum of ten people from two households were allowed to meet (religious congregations and street protests were subject to exemptions). As infection rates increased, measures were increased to a hard lockdown: private meetings were limited to five persons from two households and there were major closures of services such as schools and kindergartens, retail stores, personal care units (hairdressers, beauty salons, and similars), restaurants (with takeaway allowed), pubs, and cultural facilities. The hard lockdown lasted from December 16, 2020 until March 1, 2021, when some minor relaxations were introduced, reinstating a form of light lockdown.

### Procedure

Life with Corona is a global online survey operated by an international academic consortium. LwC was implemented to gain a better understanding of how individuals experience and cope with the COVID-19 pandemic and its countermeasures. It was launched on March 23, 2020 (first round) and revised on October 1, 2020 (second round). The survey targets adult populations (>17 years inclusion criterion) across the globe and collects data on several topics, including individual exposure to COVID-19, compliance with recommended and mandated behaviors, food security, attitudes, life satisfaction, somatic and mental health, and the sociodemographic characteristics of the respondents (age, gender, marital status, household composition, location, and living conditions). The questions about recollection of events extend to a maximum of 14 days for the mental health variables. The LwC survey can be answered in 27 languages and is promoted by local and international partners and social media. Informed consent is obtained at the beginning of the survey. The study received ethical approval by UNU-WIDER (reference number: 202009/01). More details on LwC can be retrieved online at www.lifewithcorona.org. In this paper, we use data from the second round of the survey (October 1, 2020 until February 28, 2021) from individuals who reported they live in Germany.

### Participants

Between October 1, 2020, and February 28, 2021, a total of 10,979 individuals (7,426 female; 67.6%) living in Germany completed the LwC online survey. 76.9% of answers related to the period of the hard lockdown (which started on December 16, 2020). On average, participants were 50.62 years old (SD = 16.16; range = 18–91), had 14.09 (SD = 3.99) years of formal education, and lived in a household composed of 3.03 (SD = 28.31) members. The majority of participants were either single (64.6%) or lived in a stable relationship (partner/married, 29.1%), and 52.9% lived in an urban area.

### Measures

In this subsection, we describe the data we collected and how we use them to measure the four types of GD described above.

#### Demographic Characteristics

Demographic information was collected at the beginning of the online survey, including age, years of formal education, marital status, location of residence, household composition, and gender.

#### Gender

To assess gender, we asked the participants to choose between categories of *male*, *female*, and *other*. For the analysis, we excluded participants who responded *other* due to a low response rate (only 14 participants, or 0.13%).

#### Mental Health Measures

The selected measures follow an established approach in the literature, validated in many countries, which allows comparison of results across different settings (Löwe et al., 2010; Gierk et al., 2014; Webster et al., 2014; Hinz et al., 2017).

##### Aggression

We measure aggression by applying subscales of the short version of the Buss and Perry *Brief Aggression Questionnaire* (BAQ) (Bryant and Smith, 2001; Webster et al., 2014, 2015) for physical aggression (e.g., *I have threatened people I know*; *I have trouble controlling my temper*; *Given enough provocation, I may hit another person*) and anger (e.g., *I flare up quickly but get over it quickly*; *Sometimes I fly off the handle for no good reason*; *I have trouble controlling my temper*). The instrument consists of three items for each subscale rated from *very unlike me* (1) to *very like me* (5). We calculate a sum score with values ranging 0–24, with higher values indicating a more pronounced inclination to aggression. The instrument has been applied in a wide variety of cultures (Diamond and Magaletta, 2006; Vitoratou et al., 2009; Abd-El-Fattah, 2013; Zimonyi et al., 2021), including Germany (von Collani and Werner, 2005). Webster et al. (2015) found high test–retest reliability among the four subscales indicating it measures a stable trait. Note that the instrument does not measure violent behavior but has been shown to be associated with delinquent behavior (Jurczyk and Lalak, 2020), reactive aggressive behavior in a laboratory experiment (Fahlgren et al., 2021), and aggressive acts (Archer and Webb, 2006).

##### Anxiety

We measure anxiety with subscales from the seven-item *Generalized Anxiety Disorder* scale (GAD-7) (Spitzer et al., 2006). GAD-7 has proven to be effective in assessing severity of anxiety, and it is brief and self-administered (Spitzer et al., 2006). Response options were *Not at all*; *Several Days*; *More than half days*; and *Nearly every day*, which were coded as 0, 1, 2, and 3, respectively, for each item: *Feeling nervous*, *anxious*, or *on edge*; *Not being able to stop or control worrying*; *Worrying too much about different things*; *Trouble relaxing*; *Being so restless that it’s hard to sit still*; *Becoming easily annoyed or irritable*; and *Feeling afraid as if something awful might happen*. We calculate a sum score to indicate anxiety severity, with values ranging 0–27, with higher values indicating higher anxiety. This instrument has been validated for the German population, where anxiety was correlated with low quality of life, fatigue, low habitual optimism, physical complaints, sleep problems, low life satisfaction, low social support, low education, unemployment, and low income (Hinz et al., 2017). The instrument has also been successfully applied in online surveys before (Pieh et al., 2020; Rossi et al., 2020).

##### Depression

We measure the severity of depression using the depression module of the *Patient Health Questionnaire* (PHQ-9) (Kroenke and Spitzer, 2002). All nine items of the questionnaire include rating symptoms from 0 (*not at all*) to 3 (*nearly every day*), according to the presence of a certain symptom in the 2 weeks prior to completing the survey. Based on these questions, we calculate an individual sum score that indicates depression severity. The depression score takes values ranging 0–27, with higher values indicating higher depression. The instrument has been validated for the German population (Löwe et al., 2010) and has successfully been applied as an online measure in recent studies (Pieh et al., 2020; Rossi et al., 2020).

##### Somatic symptom burden

We measure subjective severity of somatic symptoms based on the 8-item self-reported *Somatic Symptom Scale-8* (SSS8) (Gierk et al., 2014), which asks about fever, cough, diarrhea, headache, and other somatic symptoms during the 14 days before taking the survey. We calculate a sum score to indicate the symptomatic burden, taking values ranging 0–27, with higher values indicating a higher symptomatic burden. The high reliability and validity of the instrument has been demonstrated in a large sample not only in Germany (Gierk et al., 2014), but also elsewhere, including in self-administered online surveys (Matsudaira et al., 2017).

#### COVID-19 Countermeasures

To account for public policies enacted to contain the spread of the virus and the levels of life disruption that people could have experienced during the time of the study, we use the following two measures:

##### Lockdown

To contain the COVID-19 pandemic, public life in Germany was largely shut down on December 16, 2020. One week before Christmas, the hard lockdown period was implemented as the number of deaths and infections from the coronavirus reached record levels. The stringency index during the hard lockdown period reached its highest levels 83–85 (see below) For our first set of analyses, we compare the group of people that responded to the questionnaire before December 16, 2020, the start of the hard lockdown, with those that responded after that date.

##### Stringency index

The Oxford Coronavirus Government Response Tracker (OxCGRT) project (Hale et al., 2021) provides a “Stringency Index,” which indicates the strictness of public policies implemented to contain the spread of the virus. The index is calculated based on nine metrics: school closures, workplace closures, cancelation of public events, restrictions on public gatherings, closures of public transport, stay-at-home requirements, public information campaigns, restrictions on internal movements, and international travel restrictions. The resulting index is a continuous variable at the day-country level. It ranges from 1 to 100, with higher values indicating harder restrictions. We use the values of the stringency index for the time period covered in our data set, which range from 50 to 85.

#### COVID-19 Exposure

We measure COVID-19 exposure via three proxy variables, building on the measurement of shock exposure in surveys (Brück et al., 2016):

##### Testing positive for COVID-19

In the LwC survey, we asked participants if they had had an antibody coronavirus test, and whether it was positive or not.

##### Knowing someone who died

We asked survey participants whether they personally knew someone who had died from the coronavirus, or from other causes due to medical complications arising from the COVID-19 crisis.

##### Income decrease

We also asked participants if and how their monthly net income had changed since the start of the COVID-19 crisis. We group our respondents based on whether they had suffered an income loss or not.

#### Household Characteristics

We use the following three measures of household characteristics and composition:

##### Main provider

We identify those participants by asking who is the main provider of income in their household. We code the variable “yes” for participants who responded that they are the main providers and “no” if they responded that it is someone else or that they share this role with their partner.

##### Living with children

Based on information provided on other household members’ age, we group respondents into two groups: those who indicated they live in a household with children (household members below the age of 18) and those who do not.

##### Living alone

Based on information provided on the number of household members, we group respondents into two groups: those who indicated they live alone and those who do not.

### Statistical Models

We use generalized linear models (GLMs) to study GD in mental health outcomes (aggression, anxiety, depression, and symptomization) and three sets of explanatory factors: COVID-19 countermeasures, COVID-19 exposure, and household characteristics. For each factor, we included various model terms that provide interactions between a given factor with gender (two-way interactions) or with gender and another factor (three-way interactions). All models include a vector of control variables [age, years of education, location (urban or rural), and household size]. Lastly, all responses are statistically weighted based on the gender, age, education, and income distribution of the German population.

To analyze the relationship of gender and COVID-19 countermeasures with mental health outcomes, we estimate the following equation:

(1) $$H_{it}=\alpha +\beta_{1}Gender_{i}+\beta_{2}Anti-Coronameasure_{t}$$

$$+\beta_{3}Gender_{i}*Anti-Coronameasure_{t}+X_{i}+\varepsilon_{it}$$

where *H*_*it*_ refers to mental health outcome of individual *i* who answered the survey at date *t*; *Gender*_*i*_ is a dummy that equals one if the respondent was male; *Anti*−*Coronameasure*_*t*_ refers to (a) the hard lockdown indicator or (b) the stringency index; *X*_*i*_ is the vector of individual-level time-invariant control variables, and ε_*it*_ is the idiosyncratic error term. The main coefficient of interest is β_3_, which estimates how strongly the association of a COVID-19 countermeasure with a mental health outcome varies with gender.

To analyze the relationship of gender and COVID-19 exposure with mental health outcomes, we estimate the following equation:

(2) $$H_{i}=\alpha +\beta_{1}Gender_{i}+\beta_{2}Coronaexposure_{i}+$$

$$\beta_{3}Gender_{i}*Coronaexposure_{i}+X_{i}+\varepsilon_{i}$$

where *Corona exposure* is one of three separate dummy variables that indicate whether the individual (a) had had a positive result on a COVID-19 test, (b) knew someone who had died of COVID-19, or (c) had suffered an income decrease since the start of the pandemic. The main coefficient of interest is β_3_, which estimates how strongly the association of a COVID-19 exposure measure with a mental health outcome varies with gender.

To analyze how household characteristics shape the relationship of gender and anti-COVID-19 policy stringency with mental health outcomes, we estimate the following equation:

*H*_*it*_ = α + β_1_ *Gender*_*i*_ + β_2_ *Household characteristic*_*i*_ + β_3_ *Stringency index*_*t*_ + β_4_ *Gender*_*i*_ * *Household characteristic*_*i*_ + β_5_ *Gender*_*i*_ * *Stringency index*_*t*_ + β_6_ *Household characteristics*_*i*_ * *Stringency index*_*t*_ + β_7_ *Gender*_*i*_ * *Household characteristic*_*i*_ * *Stringency index*_*t*_ + *X*_*i*_ + ε_*it*_

where *Household characteristic* is one of three separate dummy variables that indicate whether the individual reported (1) being the main provider of income in her household, (2) living with children, or (3) living alone. The main coefficient of interest is β_7_, which estimates how strongly the interactive effect of a household characteristic with the stringency measures varies with gender. We conduct all tests on a significance level of at least 90%.

## Results

### Descriptives

Table 1 presents summary statistics of all variables used in the study, differentiated by gender.

**TABLE 1:** Summary statistics of all variables used in the study differentiated by gender.

	(1)	(2)	(3)	*t*-test
Variable	Female	Male	Total	Difference
	Mean/SD	Mean/SD	Mean/SD	(1)-(2)
Depression	6.043 (5.676)	5.122 (5.387)	5.612 (5.562)	0.920***
Anxiety	4.841 (5.030)	3.767 (4.527)	4.338 (4.831)	1.074***
Somatic symptom burden	5.535 (4.466)	4.228 (4.021)	4.923 (4.313)	1.306***
Aggression	2.324 (3.011)	2.517 (3.376)	2.414 (3.189)	−0.193***
Lockdown	0.764 (0.425)	0.775 (0.418)	0.769 (0.421)	−0.011
Stringency	76.969 (11.266)	77.117 (11.337)	77.038 (11.299)	−0.148
COVID-19 test positive	0.011 (0.105)	0.009 (0.096)	0.010 (0.101)	0.002
Know someone who died	0.146 (0.353)	0.131 (0.338)	0.139 (0.346)	0.015**
Income decrease	0.225 (0.417)	0.244 (0.429)	0.234 (0.423)	−0.019**
Main provider: me	0.453 (0.498)	0.626 (0.484)	0.534 (0.499)	−0.173***
Lives with children	0.235 (0.424)	0.222 (0.415)	0.229 (0.420)	0.013*
Lives alone	0.260 (0.439)	0.234 (0.423)	0.248 (0.432)	0.027***
*N*	7426	3553	10979	

The value displayed for *t*-tests are the differences in the means across the groups. ***, **, and * indicate significance at the 1, 5, and 10 percent critical level. Data is weighted.

#### Mental Health

**FIGURE 1:**
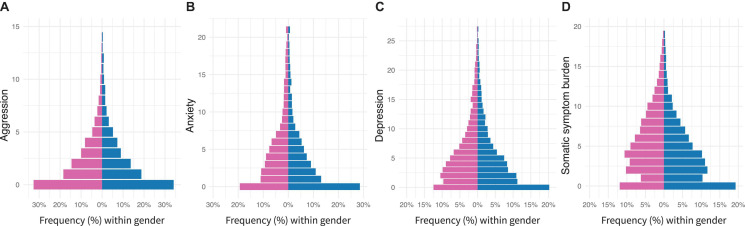
Differences between men (*n* = 3553) and women (*n* = 7426) at mental health measures: **(A)** aggression, **(B)** anxiety, **(C)** depression, and **(D)** somatic symptom burden. Gender is color-coded (blue for men). Bars represent relative percentages within gender.

The overall sum score is 5.61 (SD = 5.56) for aggression, 4.33 (SD = 4.83) for anxiety, 2.41 (SD = 3.18) for depression, and 4.92 (SD = 4.31) for somatic symptoms. *T*-tests of the difference in mean scores by gender indicate that women reported significantly higher levels for depression, anxiety, and the somatic symptom burden scores (*p* < 0.001), whereas men reported statistically higher levels of aggression (*p* < 0.001). Distributions by gender are presented in Figure 1.

#### Stringency

We do not find gender-based differences in the probability of responding before or after the lockdown and the average stringency score participants experienced was 77.04 (SD = 11.3).

#### COVID-19 Exposure

Regarding COVID-19 exposure, only 1% of the sample reported having tested positive for the coronavirus, but 13.9% knew someone who had died from COVID-19 or other causes arising from the pandemic. 23.4% of individuals had suffered an income decrease since the start of the pandemic. While there is no significant difference in the probability of reporting a positive test between genders, we find women were significantly more likely to know someone who had died of coronavirus (14.6% vs. 13.1%, *p* < 0.001), and more likely to suffer an income decrease (22.5% vs. 24.4%, *p* < 0.001).

#### Household Characteristics

With reference to household characteristics, 53.4% of the participants reported being the main income providers in their household, 22.9% of the participants live with children, and 24.8% live alone. We find women were significantly less likely to be main income providers compared to men (45.3% vs. 62.6%, *p* < 0.001); were more likely to live with children (23.5% vs. 22.2%); and were more likely to live alone (26.0% vs. 23.4%) (see Table 1).

Notably, we find variables associated with COVID-19 and household characteristics to be significantly associated with depression, anxiety, the somatic symptom burden, and aggression independently of gender (see Supplementary Material 1).

### GD in Aggression

**FIGURE 2:**
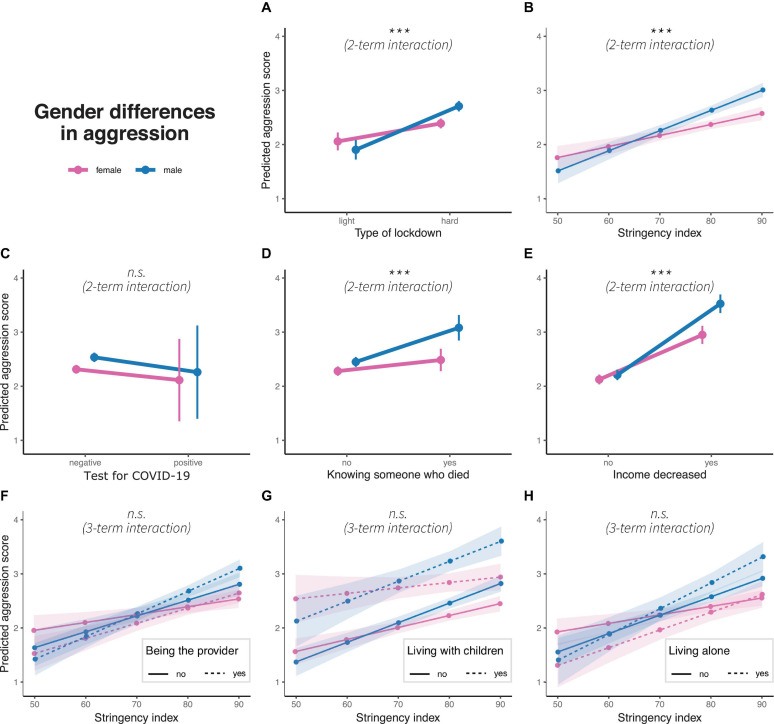
Gender differences in predicted aggression scores concerning **(A)** type of lockdown, **(B)** stringency index, **(C)** test for COVID-19, **(D)** knowing someone who died due to pandemic, **(E)** income decrease, and interaction of stringency index with **(F)** being the main household provider, **(G)** living with children and **(H)** living alone. Colors represent gender, dots represent marginal means, error bars or ribbons represent 95% confidence intervals. Linetypes represent at the bottom additional interaction terms. Significance of interaction effects are shown in italics; n.s.: non-significance. ***p < 0.01.

Generalized linear models are significant for the predictions of all aggression models (light vs. hard lockdown: *R*^2^ = 0.036, *F*(1, 10977) = 59.28, *p* < 0.01, stringency: *R*^2^ = 0.038, *F*(1, 10977) = 61.35, *p* < 0.01, positive COVID-19 test: *R*^2^ = 0.028, *F*(1, 10977) = 44.82, *p* = 0.01), knowing someone who died due to COVID-19: *R*^2^ = 0.03, *F*(1, 10977) = 48.98, *p* < 0.01), income decrease: *R*^2^ = 0.045, *F*(1, 10977) = 74.42, *p* < 0.01, being the main provider in the household *R*^2^ = 0.039, *F*(1, 10977) = 40.32, *p* < 0.01, living with children: *R*^2^ = 0.041, *F*(1, 10977) = 42.66, *p* < 0.01, and living alone: *R*^2^ = 0.039, *F*(1, 10977) = 40.61, *p* < 0.01). The positive and significant two-term interactions for light vs. hard lockdown (β = 0.47, *p* < 0.01), stringency (β = 0.02, *p* < 0.01), knowing someone who died (β = 0.46, *p* < 0.01), and income decrease (β = 0.52, *p* < 0.01) indicate increasing GD in aggression, with men consistently presenting a stronger increase in aggression than women. Two- and three-term interactions for the other variables are not significant (Figure 2). Notably, we do not find GD in aggression before the lockdown (β = −0.15, *p* < 0.21).

### GD in Anxiety

**FIGURE 3:**
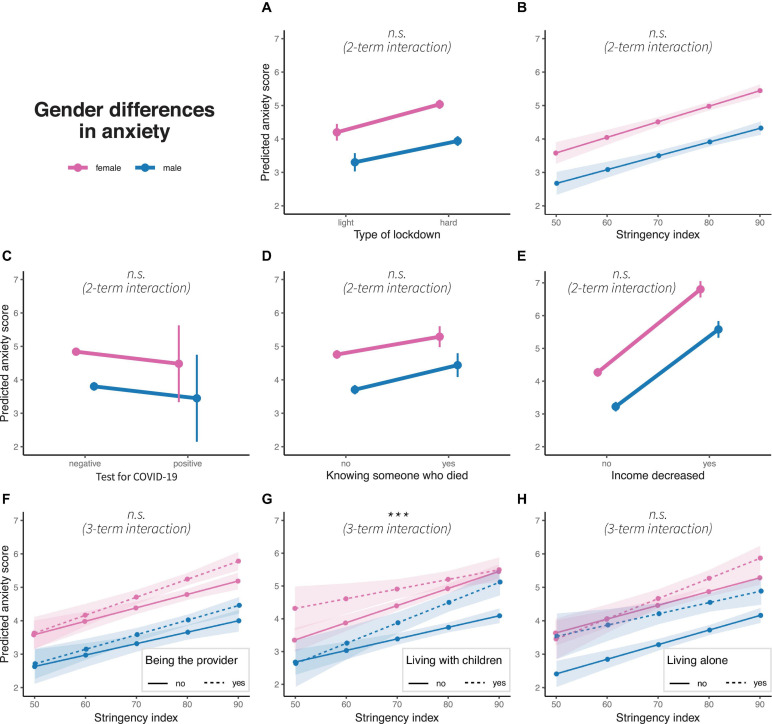
Gender differences in predicted anxiety scores concerning **(A)** type of lockdown, **(B)** stringency index, **(C)** test for COVID-19, **(D)** knowing someone who died due to pandemic, **(E)** income decrease, and interaction of stringency index with **(F)** being the main household provider, **(G)** living with children and **(H)** living alone. Colors represent gender, dots represent marginal means, error bars or ribbons represent 95% confidence intervals. Linetypes represent at the bottom additional interaction terms. Significance of interaction effects are shown in italics; n.s.: non-significance. ***p < 0.01.

Generalized linear models are also significant for all anxiety models (light vs. hard lockdown: *R*^2^ = 0.053, *F*(1, 10977) = 87.34, *p* < 0.01, stringency: *R*^2^ = 0.054, *F*(1, 10977) = 90.33, *p* < 0.01, positive COVID-19 test: *R*^2^ = 0.045, *F*(1, 10977) = 73.98, *p* = 0.01, knowing someone who died due to COVID-19: *R*^2^ = 0.047, *F*(1, 10977) = 77.92, *p* < 0.01, income decrease: *R*^2^ = 0.086, *F*(1, 10977) = 147.1, *p* > 0.01, being the main provider in the household: *R*^2^ = 0.058, *F*(1, 10977) = 61.50, *p* < 0.01, living with children: *R*^2^ = 0.055, *F*(1, 10977) = 58.32, *p* < 0.01), and living alone: *R*^2^ = 0.059, *F*(1, 10977) = 62.94, *p* < 0.01. The positive and significant three-term interaction for living with children (β = 0.49, *p* < 0.01) indicates that GD decreases when living with children and increases when living without children (see Figure 3). Other interaction terms are not significant (see Supplementary Material).

### GD in Depression

**FIGURE 4:**
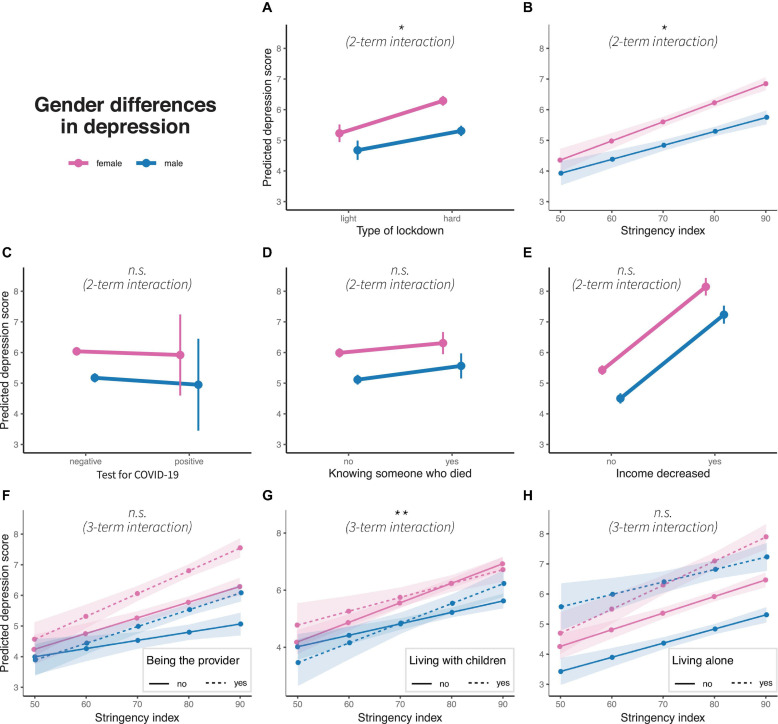
Gender differences in predicted depression scores concerning **(A)** type of lockdown, **(B)** stringency index, **(C)** test for COVID-19, **(D)** knowing someone who died due to pandemic, **(E)** income decrease, and interaction of stringency index with **(F)** being the main household provider, **(G)** living with children and **(H)** living alone. Colors represent gender, dots represent marginal means, error bars or ribbons represent 95% confidence intervals. Linetypes represent at the bottom additional interaction terms. Significance of interaction effects are shown in italics; n.s.: non-significance. *p < 0.1 and **p < 0.05.

Generalized linear models for depression models are significant for all variables (light vs. hard lockdown: *R*^2^ = 0.054, *F*(1, 10977) = 90.11, *p* < 0.01), stringency: *R*^2^ = 0.056, *F*(1, 10977) = 93.46, *p* < 0.01, positive COVID-19 test: *R*^2^ = 0.045, *F*(1, 10977) = 74.09, *p* = 0.01, knowing someone who died due to COVID-19: *R*^2^ = 0.046, *F*(1, 10977) = 75.60, *p* < 0.01, income decrease: *R*^2^ = 0.083, *F*(1, 10977) = 141.9, *p* > 0.01, being the main provider in the household: *R*^2^ = 0.065, *F*(1, 10977) = 69.77, *p* < 0.01, living with children: *R*^2^ = 0.058, *F*(1, 10977) = 61.54, *p* < 0.01, and living alone: *R*^2^ = 0.074, *F*(1, 10977) = 79.39, *p* < 0.01. Two-term interactions are significant for light vs. hard lockdown (β = −0.44, *p* < 0.1) and for stringency (β = −0.02, *p* < 0.1), both presenting increasing GD with tighter measures to control the pandemic. Three-term interactions are significant for living with children (β = 0.49, *p* < 0.05). Similar to anxiety, GD increases for individuals who do not live with children and decreases for those who live with children (see Figure 4). Other interaction terms are not significant (see Supplementary Material 2).

### GD in Somatization

**FIGURE 5:**
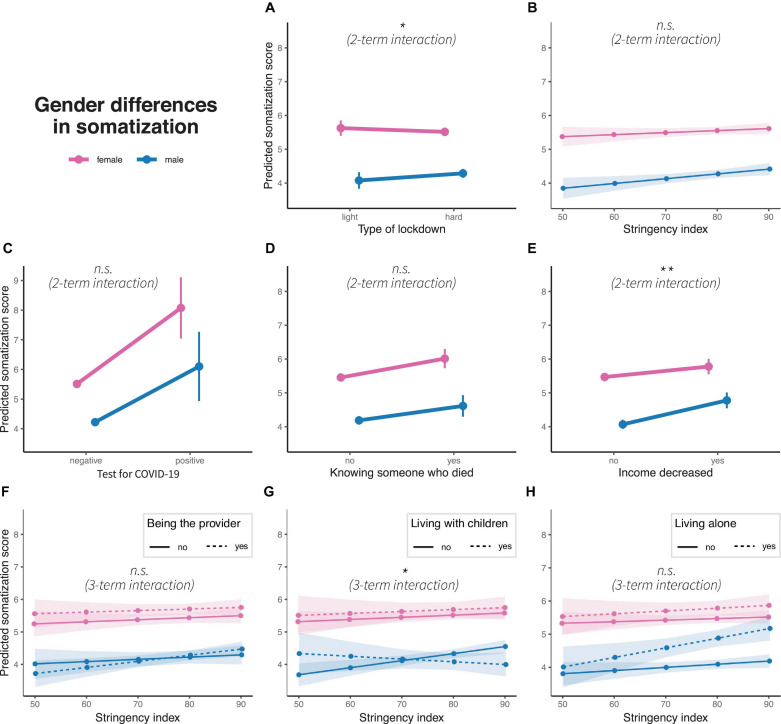
Gender differences in predicted somatic symptom burden scores concerning **(A)** type of lockdown, **(B)** stringency index, **(C)** test for COVID-19, **(D)** knowing someone who died due to pandemic, **(E)** income decrease, and interaction of stringency index with **(F)** being the main household provider, **(G)** living with children and **(H)** living alone. Colors represent gender, dots represent marginal means, error bars or ribbons represent 95% confidence intervals. Linetypes represent at the bottom additional interaction terms. Significance of interaction effects are shown in italics; n.s.: non-significance. *p < 0.1 and **p < 0.05.

Generalized linear models are also significant for the predictions of the somatic symptom burden (light vs. hard lockdown: *R*^2^ = 0.033, *F*(1, 10977) = 52.67, *p* < 0.01), stringency: *R*^2^ = 0.033, *F*(1, 10977) = 52.66, *p* < 0.01, positive COVID-19 test: *R*^2^ = 0.035, *F*(1, 10977) = 56.79, *p* = 0.01, knowing someone who died due to COVID-19: *R*^2^ = 0.034, *F*(1, 10977) = 54.43, *p* < 0.01, income decrease: *R*^2^ = 0.034, *F*(1, 10977) = 55.2, *p* < 0.01), being the main provider in the household: *R*^2^ = 0.034, *F*(1, 10977) = 34.61, *p* < 0.01, living with children: *R*^2^ = 0.034, *F*(1, 10977) = 34.97, *p* < 0.01), and living alone: *R*^2^ = 0.036, *F*(1, 10977) = 37.48, *p* < 0.01. Two-term interactions are significant for light vs. hard lockdown (β = 0.33, *p* < 0.01) and income decrease (β = 0.40, *p* < 0.05); both factors decrease GD. Three-term interaction is significant for living with children (β = −0.03, *p* < 0.1), with an increasing somatic symptom burden with increasing stringency for men living without children and a decreasing somatic symptom burden with rising stringency measures for men living with children (see Figure 5).

## Discussion

In this study, we show that GD in aggression and depression increased as a consequence of stricter COVID-19 countermeasures in Germany. While, contrary to widespread findings, men and women did not differ in their aggressiveness during the light lockdown period or when stringency was low, we find significant differences in aggressiveness during the hard lockdown and when stringency was higher. Women presented more severe depression symptoms than men, and these symptoms increased more in women than in men in periods of stricter measures. In addition, we find GD in anxiety and somatization but the results do not indicate that these increased due to COVID-19 exposure, COVID-19 countermeasures or household characteristics. We find that somatic symptom burdens increased more in men than in women during the hard lockdown, resulting in a reduction in GD. In periods of higher stringency, we find living with children to decrease GD for depression and anxiety, and to increase GD in the somatic symptom burden. GD for the group who lived without children increased with higher stringency for depression and anxiety, but GD only emerged as stringency increased. We discuss the results below in more detail for each of the four main outcomes.

### Aggression

As the stringency of the lockdown increased, men developed higher levels of aggression than women. Moreover, GD in aggression further emerged when suffering an income decrease during the pandemic (men > women) and when participants knew someone who had died due to the pandemic (men > women). GD in aggression are not significant at baseline and for household characteristics (being the main provider, living with children, living alone). While previous studies have established strong evidence for an increase of domestic and cyberviolence during the pandemic (Fraser, 2020; Perez-Vincent et al., 2020; Ebert and Steinert, 2021), we show that the pandemic facilitates the development of aggression particularly in men. Previous studies have found robust correlations of the applied aggression questionnaire with act-based violence (Archer and Webb, 2006; Jurczyk and Lalak, 2020), and emerging evidence in neuroscience points to the rewarding properties and self-perpetuating nature of violent acts (Nell, 2006; Elbert et al., 2010; Golden and Shaham, 2018; Golden et al., 2019). Additionally, gendered expectations regarding stress response further maintain GD in aggression. Our study therefore suggests that violence perpetrated by men surges during the pandemic due to the accumulating stressors and gendered expectations. Based on the emerging evidence in regard to the rewarding properties of violence, heightened levels of aggression may remain after the pandemic and the relaxation of lockdown measures. Especially online aggression, such as cyberbullying, violent video games, or the consumption/publication of other violent online material, may be novel arenas that require attention. Violent behaviors impose high costs on society in regard to executive and justice measures, psychotherapy for victims and perpetrators, and prevention programs. At the same time they might also affect public thinking and opinion when ignored (Forum on Global Violence Prevention, 2011). More research is needed to understand the magnitude of this specific consequence of the pandemic, firstly, on the individuals who experience more anger and the urge to become physically aggressive and, secondly, on the individuals who share their lives with them at work, in their family, and on social media.

### Anxiety

While our study replicates that women present more anxiety symptoms (e.g., Asher et al., 2017), we only find GD developing differently for men and women who lived with vs. without children. Here, the GD in individuals who lived with children are significant when stringency was low, but disappear–with men developing similarly high levels of depression as women–when they lived with children. In contrast, GD are not significant when stringency was low, but increase for men and women who lived without children with more stringent measures. Other interaction terms that we test are not significant. The latter is in line with the study of Ausín et al. (2021) from Spain. Following the rationale in the introduction, we would have expected women to be biologically more prone to develop, socially more prepared to express and in terms of gender roles more vulnerable to present higher levels of anxiety. This is not supported by our results, except for women who live without children, as they present a steeper increase of anxiety than men who live without children. In contrast, men who live with children seem to adopt “female levels of anxiety.” We did not expect this. What happens to the fathers who live with children during heightened stringency levels? One explanation may be that men faced high pressure both at work and as providers while having to arrange work from home or sharing responsibility for their children with their partner. There might also be a gender gap in the extent to which it is accepted for men to fail on tasks due to childcare. In turn, this could have allowed some relief to women who were not able to perform due to homeschooling and quarantines. Higher levels of empathy devoted to women might have facilitated this development. However, men and women respond equally to the crisis in terms of anxiety in general, though both showing an increase.

### Depression

The lockdown and higher stringency measures are associated with higher levels of depression (replicating e.g., ref) and an increased GD in depression. According to our expectations, women developed higher levels of depression during the hard lockdown or when stringency increased, respectively. The findings are partly in contrast to the study of Ausín et al. (2021) from Spain who found that GD in depression was not increasing there.

As for anxiety, when living with or without children we find a more complex pattern of GD: no significant differences during the low stringency period in individuals without children but a steeper increase for women than for men when stringency increased. For individuals who reported living with children, we find a trend toward assimilating levels of depression in men when stringency levels intensified. We also find similar levels of depression for men and women who lived alone. This contradicts previous theories concerning the consequences of combining multiple roles (spouse, parent, and worker) on mental health with a stronger impact on women than men (Simon, 1995). Our result indicates that men are strongly affected by stricter stringency when they live alone or with children. Moreover, this contrasts with previous studies that found having children to constitute a protective factor for mental ill-health for men only, with men who had two or more children presenting a lower risk of developing mental disorders when compared to men without children (Klose and Jacobi, 2004).

On the other hand, the result that GD in mental disorders increased for women is in line with other COVID-19 studies that reported a new gap in psychological distress emerging between women with children at school age and women without children (Zamarro and Prados, 2021). Similarly, Mazza et al. (2020) suggested that women who lived alone or had children with behavioral problems may present an increase of mental disorders, such as stress or depression. An emerging research gap that results from this study is in regard to men who develop more depression in their dual role as fathers at home with children and providers. Does sharing responsibility at work and in the household mean that the burden is doubled and that both partners have to carry their weight? Or can the burden actually be shared?

Hard lockdown and increasing stringency are associated with a steeper increase in depression for women than for men. One factor that may contribute to this are the heightened levels of domestic and gender-based violence (GBV) documented during the lockdowns. Indeed, this has previously been claimed to be a major health concern during the pandemic (Bradbury-Jones and Isham, 2020; Telles et al., 2020), who argued that the impact of the pandemic has fueled stress and tensions within families, with an increased and continuous risk of domestic violence and divorce cases (Chang, 2020; Peterman et al., 2020; Usher et al., 2020). Accordingly, several studies reported that the increase of domestic violence occurred especially during stricter lockdowns or states of emergency (Campbell, 2020; Usher et al., 2020), e.g., a 55% increase of calls made to a domestic violence hotline during the lockdown in Argentina (Perez-Vincent et al., 2020), or the reported increased risk of domestic violence during the pandemic in Germany (Ebert and Steinert, 2021).

Furthermore, evidence shows that family violence, including IPV, child abuse and elder abuse, increase during and after large-scale crises or disasters (Neria et al., 2008; Perez-Vincent et al., 2020; Peterman et al., 2020). Families from socially deprived settings and with low socioeconomic status (e.g., low educational levels or unemployment) are particularly at risk in Germany (Ravens-Sieberer et al., 2021). Women and children are most affected by these incidents globally and, although data is still scarce, the domestic violence rates appear to be rising rapidly (Perez-Vincent et al., 2020). Yet, children and adolescents in Germany do not seem to be as negatively affected as, for example, youth from Spain and Italy, where higher levels of stress on families were observed, with 85.7% of the parents reporting emotional and behavioral changes in their children (Orgilés et al., 2020); or in China, where one study reported 22.6% of students having depressive symptoms, which is higher than the 17.2% previously reported in studies on primary schools (Xie et al., 2020).

In addition to GBV, a combination of the biologically determined heightened susceptibility of women for depressive symptoms (e.g., Slavich and Sacher), combined with gender-specific roles that come with restrictions and opportunities (Seedat et al., 2009; Salk et al., 2017), may underlie the enforced GD during periods of high-stringency lockdown measures. However, it is unclear why lockdowns and stringency do not increase GD in anxiety but only in depression. Both syndromes are more pronounced in women than in men and related to adversity (Kuzminskaite et al., 2021). One explanation, yet speculative, could be that the pandemic imposes a situation with both an invisible or implicit threat and also highly incisive inescapable consequences, connected with the uncertainty regarding what the post-pandemic period will look like and when it will start.

To the best of our knowledge, there is no study that investigates characteristics of threats in association with specific symptoms of depression and anxiety. However, it is known that emotional abuse and neglect (especially during childhood) may lead to more pronounced symptoms of depression than post-traumatic stress or anxiety. Indeed, the pandemic has imposed strong restrictions on meeting family, friends, and other community members. Accordingly, living alone has been shown to be a consistent moderator of symptom severity, both in anxiety and depression. The social deprivation may therefore have had a stronger weight than the actual threat to physical integrity. From a sociological perspective, these results indicate that, when traditional resources of social cohesion are interrupted (real life sociality), a substantial part of resilience may be undermined.

But why is there a difference by gender? One explanation may be the gendered labor patterns in Germany which are outlined in detail below in the section “Moderating Social Factors Associated With the COVID-19 Pandemic.” In summary, it means that, on average, men engage more in paid work while women remain occupied in informal care and part-time jobs–or poorly paid jobs, e.g., in supermarkets or as nurses. Since the German government avoided the wholesale closure of industries, men were less likely to be confined to their homes and thus able to socialize with their colleagues at work, whereas many retail businesses and the hospitality industry were shut. As female employment is relatively high in these sectors, more women were cut off from their social environments and isolated with their children at home.

Many of the children themselves had difficulties coping and developed abnormal behaviors (Wang G. et al., 2020), which impacted their parents (Calvano et al., 2021)–mothers and fathers alike–as indicated by our results. This is where the pandemic comes down to the concept of Seligman’s learned helplessness from about four decades ago. Many people have realized that there is no escape from this crisis. Policies and stepped care mental health programs could provide relief and help people reestablish social activities, regain a feeling of autonomy (external rather than internal), reprocess and contextualize the most important events (that the pandemic was a special situation requiring special measures rather than a global plan to serve the self-interest of anyone), and to close off the period (to counteract the attribution that the pandemic is a permanent condition).

### Somatization

The overall subjective burden of somatic symptoms was higher in women than in men. Yet, the hard lockdown period, an income decrease, and higher-stringency lockdown measures resulted, for participants who lived with children, in a steeper increase of somatic symptoms in men than in women (decreasing GD). Increased vulnerability of men in developing severe symptoms after a COVID-19 infection have been well established (Conti and Younes, 2020; Jin et al., 2020; Wenham, 2020). However, our study does not find a higher level of somatic symptoms in men for those who tested positive. On the other hand, our sample includes only a very small percentage of participants who tested positive for COVID-19. Given the potential long-term consequences of COVID-19 infections and the consistent association of the somatic symptoms with mental health problems, further research would be beneficial.

In the case of men who lived with children assimilating to the somatic symptom burden of women, it is noteworthy that the COVID-19 crisis does not actually seem to have increased GD in terms of levels of parental involvement in childcare. In Germany, for example, studies observed that both parents reported spending substantially more time with their children during the crisis than they did in the previous year (Kreyenfeld et al., 2020), and there are seemingly no elementary differences in established aggregate-level roles of division of labor in couples (Hank and Steinbach, 2020).

### Moderating Social Factors Affected by the COVID-19 Pandemic

#### Paid and Non-paid Work

There is considerable evidence showing an overall magnifying of gender inequalities in paid and non-paid work during COVID-19 (Farré et al., 2020). Although women are as likely as men to have flexible jobs, women globally earn less than men and were already in a more vulnerable situation before the pandemic started (Carli, 2020). The pandemic increases this inequality, because women disproportionately occupy a share of jobs requiring face-to-face interactions, e.g., retail or personal care, meaning that the opportunities to work from home and the risk of unemployment are higher (Freund and Hamel, 2020). Moreover, an important share of women are essential workers (Boniol et al., 2019), e.g., healthcare workers, with an increased risk of infection from the coronavirus, putting them at a higher risk of stress and burnout (Carli, 2020).

#### Domestic Division of Labor

In many OECD countries, including Germany, the domestic division of labor still predominantly follows a traditional system (Zimmert, 2019). Despite recent policy reforms that have resulted in some increase in maternal full-time work and an increase in fathers taking parental leave, equally shared care work among both partners is still the exception, especially in West Germany (Hank and Steinbach, 2020; Kreyenfeld et al., 2020). The same is observed in the United Kingdom, with mothers spending less time in paid work but more time on household responsibilities (mothers combined paid work with other activities–mostly childcare–47% of their time, compared with 30% for fathers, a 2:1 ratio) (Andrew et al., 2020). Similar results are reported in Spain, with mothers spending on average 28 h a week on childcare compared with 19 h for fathers (Farré et al., 2020) see also (Czymara et al., 2021) for further discussion).

#### Informal Care

COVID-19 and its countermeasures have impacted women and men differently in terms of family dynamics and the intra-household allocation of paid and family care work, with consequences for both physical and mental health. Gender norms fundamentally shape women’s and men’s lives and this pandemic has remarkably increased the need for care inside homes, which has a particularly large impact on working mothers (Alon et al., 2020). Women underpin a greater share of informal care, providing on average 3.3 times more care than men at home (Addati and Cattaneo, 2018; Manzo and Minello, 2020), with the consequence of limiting their work and economic opportunities (Wenham, 2020). Comprehensively, social isolation was found to affect women in particular, considering that, for example, school closures forced more women than men to take time off for childcare, which might provide some insights into women’s loneliness and depression (Chang, 2020). Thus, for the past three decades, studies have consistently reported that women are more likely to experience depression than men (Salk et al., 2017; Weissman and Klerman, 1977). However, as Brommelhoff et al. (2004) observes, reporting bias might also contribute to the higher rates of depression in women, since even when men and women present similar depression symptoms, women are tendentiously more likely to be diagnosed with depression, which might underlie a gender bias. In line with this, Bluhm (2011) argues that depressed women do not tend to simply act out passive behavior, but are more aware of their symptoms, despite the effects of their environment and their illness.

### Insight and Implications

Our study highlights unresolved questions for research and policy. The first one regards aggression. How can increasing aggression in men be addressed effectively during and after the pandemic? Barriers to seek services as a victim, but also as a perpetrator, have to be distinguished into community and online programs. More research is necessary to estimate the demand during pandemics and adequately scale up these services. Furthermore, the focus in research and practice should shift toward prevention programs for perpetrators, since they may be at particular risk for developing a robust trait of (appetitive) aggression, antisocial personality disorder, or psychopathy (Powell et al., 1997; Cauffman et al., 1998; Fondacaro et al., 1999; Garieballa et al., 2006). Thus, screening for traumatic events should become a routine part of psychological treatment. Combined trauma therapy with extended group sessions allowing for skills training and help to abstain from aggression has shown promising results (Robjant et al., 2019; Koebach et al., 2021). In addition, systemic approaches help to de-escalate intrinsic family dynamics that lead to aggression and violence (e.g., Oka and Whiting, 2011). However, prevention should start with the adequate care of trauma-exposed children and youths, especially young men who learn to experience and control their aggression as part of their developmental milestones (Kröber, 2012).

Second, our data show that individuals living with children–male or female–present higher levels of depression and anxiety. Due to changes in the workplace and within families, more men will have to juggle paid work with housework. This will lead to higher stress loads and the question of how families can be protected. The increased stress load during the pandemic highlights the importance of well-functioning childcare services and schools, not only as a measure to raise and prepare the next generation for the workplace but also to facilitate mental health in society. Further research should explore the clinical relevance of the symptoms and whether gender assimilation in depression is maintained and how it develops after the pandemic. More research is needed to explore the specific challenges faced by men during the pandemic (see also Betron et al., 2020). It is also important to note that children growing up with a depressed parent have a higher risk of developing mental disorders themselves (Downey and Coyne, 1990).

Third, the higher levels of depression in women during crises lead to the question of whether depression in women can be prevented through gender equality programs. Programs to assist women to cope with the additional stress may be particularly important. In a large multinational study (Seedat et al., 2009) indeed found a decrease in GD in depression for countries with more gender equality. Given the hormonal aspects in the development of depression, this should nevertheless be complemented with early psychotherapeutic intervention. Mental health problems induced by the pandemic are likely to persist over time and mental health services should prepare for higher numbers of patients emerging after the COVID-19 pandemic. Currently, the demand for mental healthcare is rapidly increasing, but affected individuals experience higher barriers to care due to lockdown measures being in place and services being overstretched. According to a WHO survey of 130 member states launched in mid-June 2020, the COVID-19 pandemic disrupted or halted critical mental healthcare in 93% of these countries, with approximately only 30% of mental health services for children, adolescents or older adults reporting no disruptions (WHO, 2020).

Fourth, depression and anxiety are higher in individuals who live alone, leading to the question how we can improve their resilience. Targeted regulations and interventions are necessary to shield this population from the negative mental health effects. Again, it will be an essential question how many suffer at a clinically relevant level and what the recovery rate is after the crisis. A model example is the project *Coping with Corona: Extended Psychosomatic care in Essen* (CoPE) developed to target, prevent and address the psychological burden of the pandemic, via a community-based intervention. CoPE aims at providing health support with psychoeducation, mindfulness and cognitive behavioral skills training. The intervention addresses day structuring, fears and worries, conflicts, stress management, sleep, and loneliness (Bäuerle et al., 2020c).

Fifth, despite the diversity in individual experiences, COVID-19 has affected everyone. Community intervention may therefore help a collective reprocessing of the pandemic and thus prevent further divisions in society. The key question is whether there is a desire and a political will to form a collective memory. The pandemic has led to divergent adverse, sometimes traumatic experiences of the crisis in terms of age/generation, gender, socioeconomic status, and work group (e.g., for medical doctors and nurses). Immediate responses tend to avoid and close with the past while ignoring the personal wounds and societal cleavages that were generated. In the long run, this may divide society. Therefore, a collective process to restore a shared understanding of the pandemic is necessary to rebuild a sense of togetherness and community. To this end, the narratives of subgroups who experienced marginalization and disadvantage due to policies related to the pandemic can be merged and developed to be presented to the affected community and facilitate a shared collective memory of the living generation (Koebach and Robjant, sub).

### Strengths and Limitations

Our study has several strengths. First, it is one of the largest samples to date to examine GD in mental health burdens globally and in Germany during COVID-19. Second, besides mental health issues that were known to be more common in women, we also investigate aggression as an important mental health outcome. Third, LwC collects data in real time, reducing memory bias and allowing a valid comparison on the impact of the introduction or relaxation of COVID-19 countermeasures over time. Fourth, we collect nuanced information on what we call the exposure to COVID-19, which covers both respondents’ own health experience of the pandemic, that of their social circle and an economic dimension. Finally, to our knowledge, there is no previous study that investigated gender effects in somatization dependent on the pandemic.

Nevertheless, our study also has some important limitations. First, the sampling technique we used to collect data is an online survey and we have to consider the possibility of selection bias, as suggested by the unbalanced gender ratio observed, e.g., when comparing cross-sectional data during the light and hard lockdown periods, which are based on answers by different individuals. Even though we statistically weighted sample responses based on population data on gender, age, level of education, and income, questions about representativeness and comparability over time may remain. Second, the responses were based on a self-administered online survey, which might create systematic differences in answers compared to answers from in-person interviews (though responses may suffer less from enumerator bias). Third, the variable whether the participant had previously contracted a COVID-19 infection is also a self-report rather than based on a test result. Fourth, we have limitations concerning sociodemographic data. since this is an international survey and the constructs of ethnicity and race are culturally specific, LwC does not include any questions on ethnicity or race. Also, LwC only includes a gender variable as: *male*, *female* or *other*, and does not include non-binary gender identity considerations. We acknowledge that a binary division of gender has been called into question and a more fluid and inclusive understanding of gender should be developed. Finally, our sample consisted of a very small part of individuals who tested positive for COVID-19 which restricts the relevance of the non-significant results and requires further investigation, possible using drawing on more data from the LwC survey or similar datasets.

## Data Availability Statement

The original contributions presented in the study are included in the article/Supplementary Material, further inquiries can be directed to the corresponding author/s.

## Ethics Statement

The studies involving human participants were reviewed and approved by UNU-WIDER (reference number: 202009/01). The patients/participants provided their written informed consent to participate in this study.

## Author Contributions

LA and AK made substantial contributions to the conception or design of the work and drafting the work. AH, TB, WS, and PJ made substantial contributions to the conception and design and were project leaders of Life with Corona (LwC). LA, AK, OD, and SC made substantial contributions to the analysis or interpretation of data for the work. LA, AK, OD, SC, AH, WS, HF, PJ, and TB made substantial contribution to the revision of the analysis and interpretation of data for the work and revising it critically for important intellectual content. LA, AK, OD, SC, AH, WS, HF, PJ, and TB provided approval for publication of the content and agreed to be accountable for all aspects of the work in ensuring that questions related to accuracy of any part of the work are appropriately investigated and resolved. All the authors contributed and approved the submitted version of this article.

## Conflict of Interest

The authors declare that the research was conducted in the absence of any commercial or financial relationships that could be construed as a potential conflict of interest.

## Publisher’s Note

All claims expressed in this article are solely those of the authors and do not necessarily represent those of their affiliated organizations, or those of the publisher, the editors and the reviewers. Any product that may be evaluated in this article, or claim that may be made by its manufacturer, is not guaranteed or endorsed by the publisher.
