# Supplementary material for: Life With Corona: Increased Gender Differences in Aggression and Depression Symptoms Due to the COVID-19 Pandemic Burden in Germany
Source: Front Psychol. 2021 Jul 27;12:689396. doi: 10.3389/fpsyg.2021.689396 (PMC8353131; doi:10.3389/fpsyg.2021.689396)

Supplementary Material

Paper submission to Frontieres of Psychology

Special Issue “Gender Differentials in Times of COVID-19”

Deadline: 28 May 2021

**Life with Corona: increased gender differences in depression symptoms and aggressiveness due to the pandemic burden in Germany**

**Liliana Abreu**^1*,†^**, Anke Koebach**^2,†^**, Oscar Díaz**^3^**, Samuel Carleial**^2^**, Anke Hoeffler**^1^**, Wolfgang Stojetz**^3^**, Hanna Freudenreich**^4^**, Patricia Justino**^5^**, Tilman Brück**^3,4,6^

^1^Development Research Group, Department of Politics and Public Administration, University of Konstanz, Konstanz, Germany

^2^Clinical and Neuropsychology, Department of Psychology, University of Konstanz, Konstanz, Germany

^3^ISDC - International Security and Development Center, Berlin, Germany

^4^Leibniz Institute of Vegetable and Ornamental Crops, Großbeeren, Germany

^5^World Institute for Development Economic Research, United Nations University, Finland

^6^Natural Resources Institute, University of Greenwich, Chatham Maritime, United Kingdom

*** Correspondence:**

Liliana Abreu

liliana.abreu@uni-konstanz.de

† These authors have contributed equally to this work and share first authorship.

**Keywords: depression, aggression, anxiety, somatization, mental health, COVID-19 pandemic, gender differences.**

Abbreviation

GD = Gender difference

# Descriptive statistic of measures and mental health in Germany

|  | Aggression | | | Anxiety | | | Depression | | | Somatic symptoms burden | | | N | |
| --- | --- | --- | --- | --- | --- | --- | --- | --- | --- | --- | --- | --- | --- | --- |
|  | (1) | (2) | t-test | (1) | (2) | t-test | (1) | (2) | t-test | (1) | (2) | t-test | (1) | (2) |
| Variable | No | Yes | Difference | No | Yes | Difference | No | Yes | Difference | No | Yes | Difference | No | Yes |
|  | Mean | Mean | (1)-(2) | Mean | Mean | (1)-(2) | Mean | Mean | (1)-(2) | Mean | Mean | (1)-(2) |  |  |
| Lockdown | 1.988 | 2.542 | -0.555*** | 3.791 | 4.502 | -0.712*** | 4.977 | 5.803 | -0.825*** | 4.92 | 4.924 | -0.004 | 3173 | 7806 |
|  | (2.673) | (3.317) |  | (4.211) | (4.991) |  | (4.990) | (5.708) |  | (4.046) | (4.390) |  |  |  |
| Corona test positive | 2.416 | 2.234 | 0.182 | 4.34 | 4.133 | 0.207 | 5.612 | 5.61 | 0.002 | 4.898 | 7.344 | -2.446*** | 10860 | 119 |
|  | (3.194) | (2.603) |  | (4.836) | (4.385) |  | (5.565) | (5.201) |  | (4.282) | (6.217) |  |  |  |
| Know someone who died | 2.365 | 2.72 | -0.355*** | 4.252 | 4.869 | -0.617*** | 5.561 | 5.927 | -0.365** | 4.854 | 5.354 | -0.501*** | 9431 | 1548 |
|  | (3.133) | (3.499) |  | (4.776) | (5.127) |  | (5.515) | (5.834) |  | (4.244) | (4.694) |  |  |  |
| Income decreased | 2.168 | 3.224 | -1.057*** | 3.774 | 6.187 | -2.413*** | 4.983 | 7.677 | -2.694*** | 4.817 | 5.271 | -0.453*** | 8512 | 2467 |
|  | (2.918) | (3.839) |  | (4.368) | (5.734) |  | (5.123) | (6.385) |  | (4.226) | (4.570) |  |  |  |
| Main provider: me | 2.372 | 2.452 | -0.08 | 4.229 | 4.433 | -0.205** | 5.261 | 5.918 | -0.657*** | 4.953 | 4.897 | 0.056 | 5294 | 5685 |
|  | (2.979) | (3.360) |  | (4.731) | (4.916) |  | (5.290) | (5.771) |  | (4.312) | (4.314) |  |  |  |
| Lives with children | 2.25 | 2.968 | -0.718*** | 4.208 | 4.775 | -0.567*** | 5.564 | 5.773 | -0.209* | 4.913 | 4.957 | -0.044 | 8042 | 2937 |
|  | (3.089) | (3.447) |  | (4.840) | (4.777) |  | (5.572) | (5.526) |  | (4.345) | (4.203) |  |  |  |
| Lives alone | 2.416 | 2.41 | 0.006 | 4.2 | 4.757 | -0.557*** | 5.248 | 6.717 | -1.469*** | 4.79 | 5.327 | -0.536*** | 8258 | 2721 |
|  | (3.140) | (3.333) |  | (4.725) | (5.117) |  | (5.355) | (6.013) |  | (4.227) | (4.541) |  |  |  |

The value displayed for t-tests are the differences in the means across the groups. ***, **, and * indicate significance at the 1, 5, and 10 percent critical level.

# GLMs regression tables and figures

## GD in mental health before and after the lockdown

|  | (1) | (2) | (3) | (4) |
| --- | --- | --- | --- | --- |
| VARIABLES | Aggression | Anxiety | Depression | Somatic symptom burden |
|  |  |  |  |  |
| Gender: Male | -0.156 | -0.897*** | -0.554** | -1.548*** |
|  | (0.125) | (0.188) | (0.216) | (0.169) |
| Lockdown | 0.486*** | 1.161*** | 1.529*** | 0.083 |
|  | (0.100) | (0.150) | (0.172) | (0.135) |
| Male x Lockdown | 0.478*** | -0.204 | -0.436* | 0.328* |
|  | (0.142) | (0.214) | (0.246) | (0.193) |
|  |  |  |  |  |
| Observations | 10,979 | 10,979 | 10,979 | 10,979 |
| R-squared | 0.036 | 0.053 | 0.054 | 0.032 |
| Model | Linear | Linear | Linear | Linear |
| F(1, 10977) | 59.22 | 87.05 | 89.87 | 52.61 |

Standard errors in parentheses *** p<0.01, ** p<0.05, * p<0.1 *Lockdown* is a variable that indicates whether the person responded the survey after the beginning of the lockdown (16 December 2020).

*Controls*: age, household size, years of education, urban vs rural; data was weighted on age, gender and level of education.


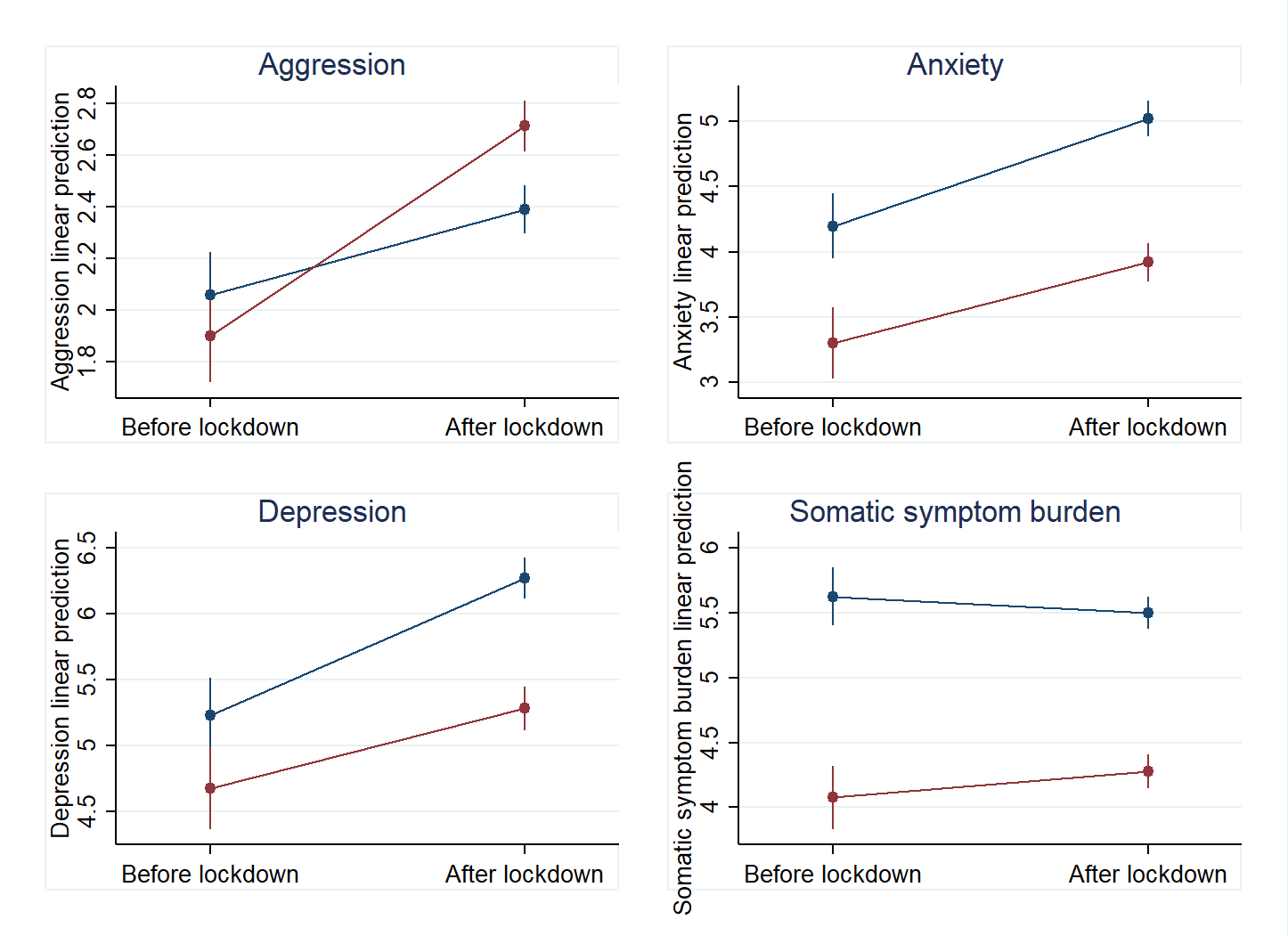


## GD in mental health and stringency of measures to control the spread of the virus

|  | (1) | (2) | (3) | (4) |
| --- | --- | --- | --- | --- |
| VARIABLES | Aggression | Anxiety | Depression | Somatic symptom burden |
|  |  |  |  |  |
| Gender: Male | -1.087*** | -0.637 | 0.409 | -1.927*** |
|  | (0.412) | (0.619) | (0.712) | (0.559) |
| Stringency | 0.020*** | 0.047*** | 0.062*** | 0.006 |
|  | (0.004) | (0.006) | (0.006) | (0.005) |
| Male x Stringency | 0.017*** | -0.005 | -0.017* | 0.008 |
|  | (0.005) | (0.008) | (0.009) | (0.007) |
|  |  |  |  |  |
| Observations | 10,979 | 10,979 | 10,979 | 10,979 |
| R-squared | 0.038 | 0.054 | 0.056 | 0.033 |
| Model | Linear | Linear | Linear | Linear |
| F(1, 10977) | 61.35 | 90.33 | 93.46 | 52.66 |

Standard errors in parentheses *** p<0.01, ** p<0.05, * p<0.1 *Stringency* is a variable that indicates the stringency of anti-Corona measures that day of the individual filled in the survey.

*Controls*: age, household size, years of education, urban vs rural; data was weighted on age, gender and level of education.


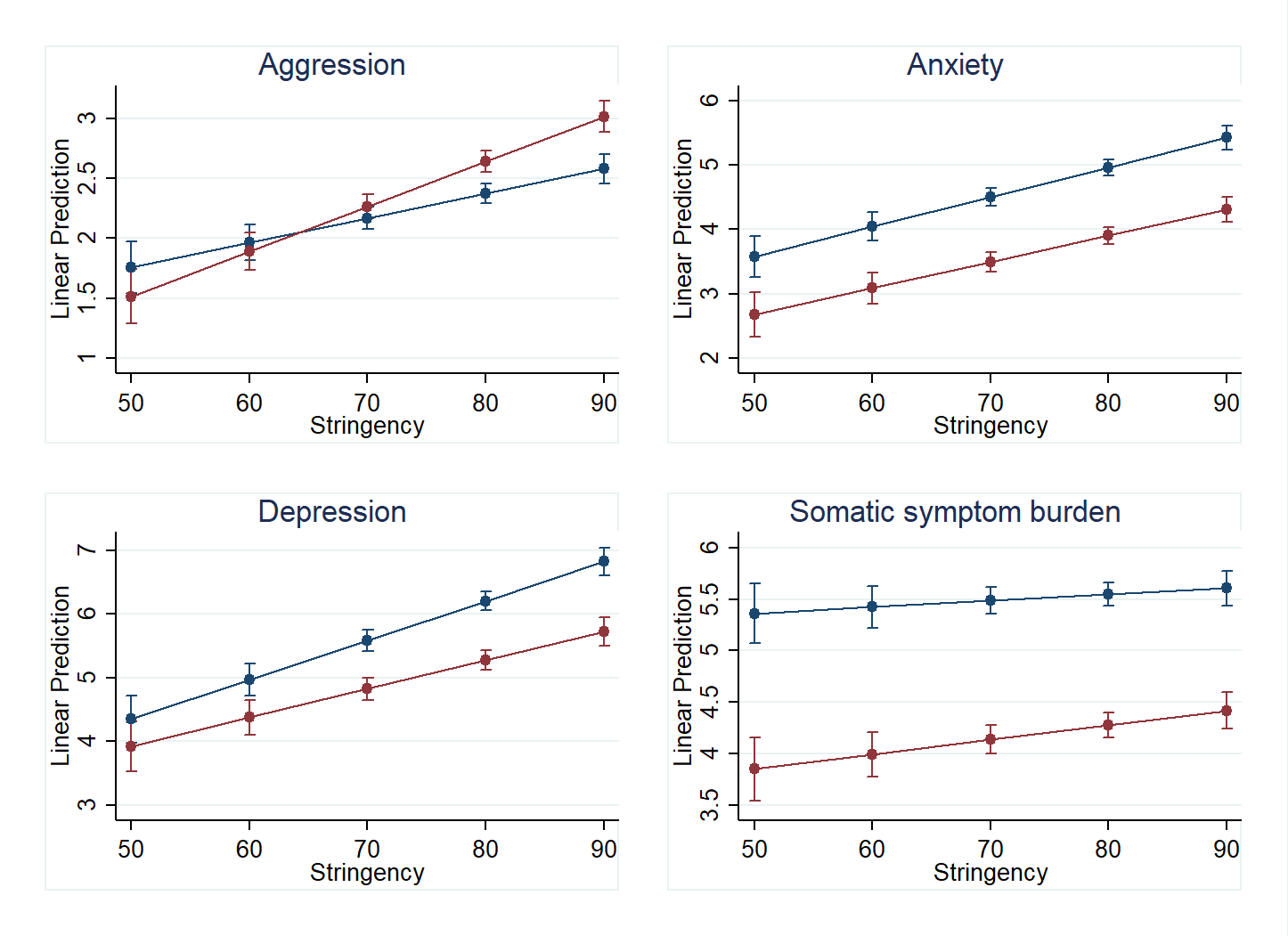


## GD in mental health and corona exposure (someone known died due to the pandemic)

|  | (1) | (2) | (3) | (4) |
| --- | --- | --- | --- | --- |
| VARIABLES | Aggression | Anxiety | Depression | Somatic symptom burden |
|  |  |  |  |  |
| Gender: Male | 0.222*** | -1.040*** | -0.871*** | -1.282*** |
|  | (0.061) | (0.091) | (0.105) | (0.082) |
| Corona test positive | -0.194 | -0.339 | -0.084 | 2.586*** |
|  | (0.391) | (0.587) | (0.676) | (0.527) |
| Male x Corona test positive | 0.043 | 0.168 | 0.096 | -0.460 |
|  | (0.601) | (0.902) | (1.039) | (0.810) |
|  |  |  |  |  |
| Observations | 10,979 | 10,979 | 10,979 | 10,979 |
| R-squared | 0.028 | 0.045 | 0.045 | 0.035 |
| Model | Linear | Linear | Linear | Linear |
| F(1, 10977) | 44.82 | 73.98 | 74.09 | 56.79 |

Standard errors in parentheses *** p<0.01, ** p<0.05, * p<0.1

*Controls*: age, household size, years of education, urban vs rural; data was weighted on age, gender and level of education.


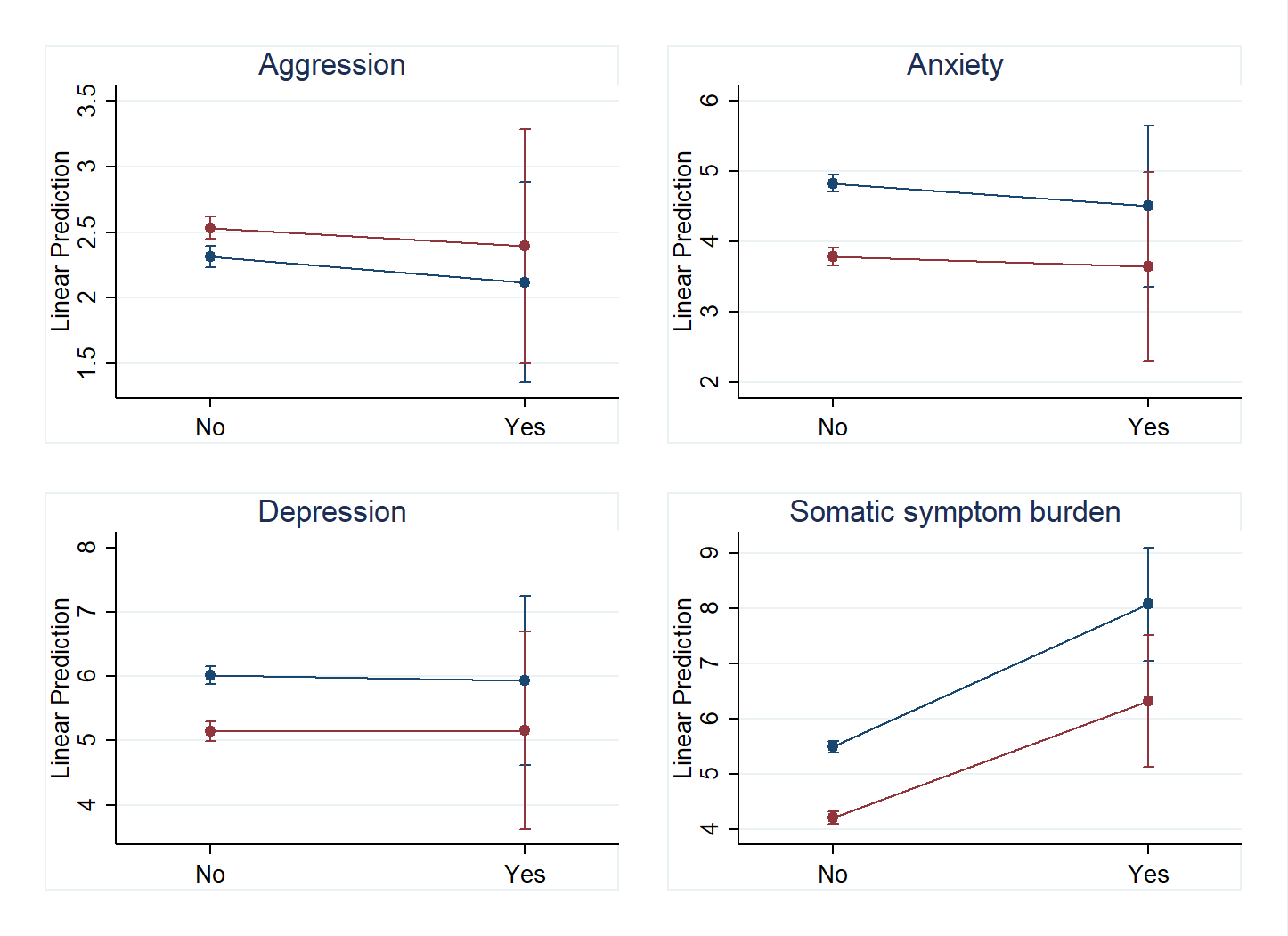


## GD in mental health and corona exposure (someone known died due to the pandemic)

|  | (1) | (2) | (3) | (4) |
| --- | --- | --- | --- | --- |
| VARIABLES | Aggression | Anxiety | Depression | Somatic symptom burden |
|  |  |  |  |  |
| Gender: Male | 0.166** | -1.063*** | -0.888*** | -1.282*** |
|  | (0.065) | (0.097) | (0.112) | (0.087) |
| Know someone who died | 0.204* | 0.548*** | 0.389* | 0.528*** |
|  | (0.117) | (0.175) | (0.202) | (0.157) |
| Male x Know someone who died | 0.455*** | 0.257 | 0.182 | -0.007 |
|  | (0.174) | (0.262) | (0.302) | (0.235) |
|  |  |  |  |  |
| Observations | 10,979 | 10,979 | 10,979 | 10,979 |
| R-squared | 0.030 | 0.047 | 0.046 | 0.034 |
| Model | Linear | Linear | Linear | Linear |
| F(1, 10977) | 48.98 | 77.92 | 75.60 | 54.43 |

Standard errors in parentheses *** p<0.01, ** p<0.05, * p<0.1

*Controls*: age, household size, years of education, urban vs rural; data was weighted on age, gender and level of education.


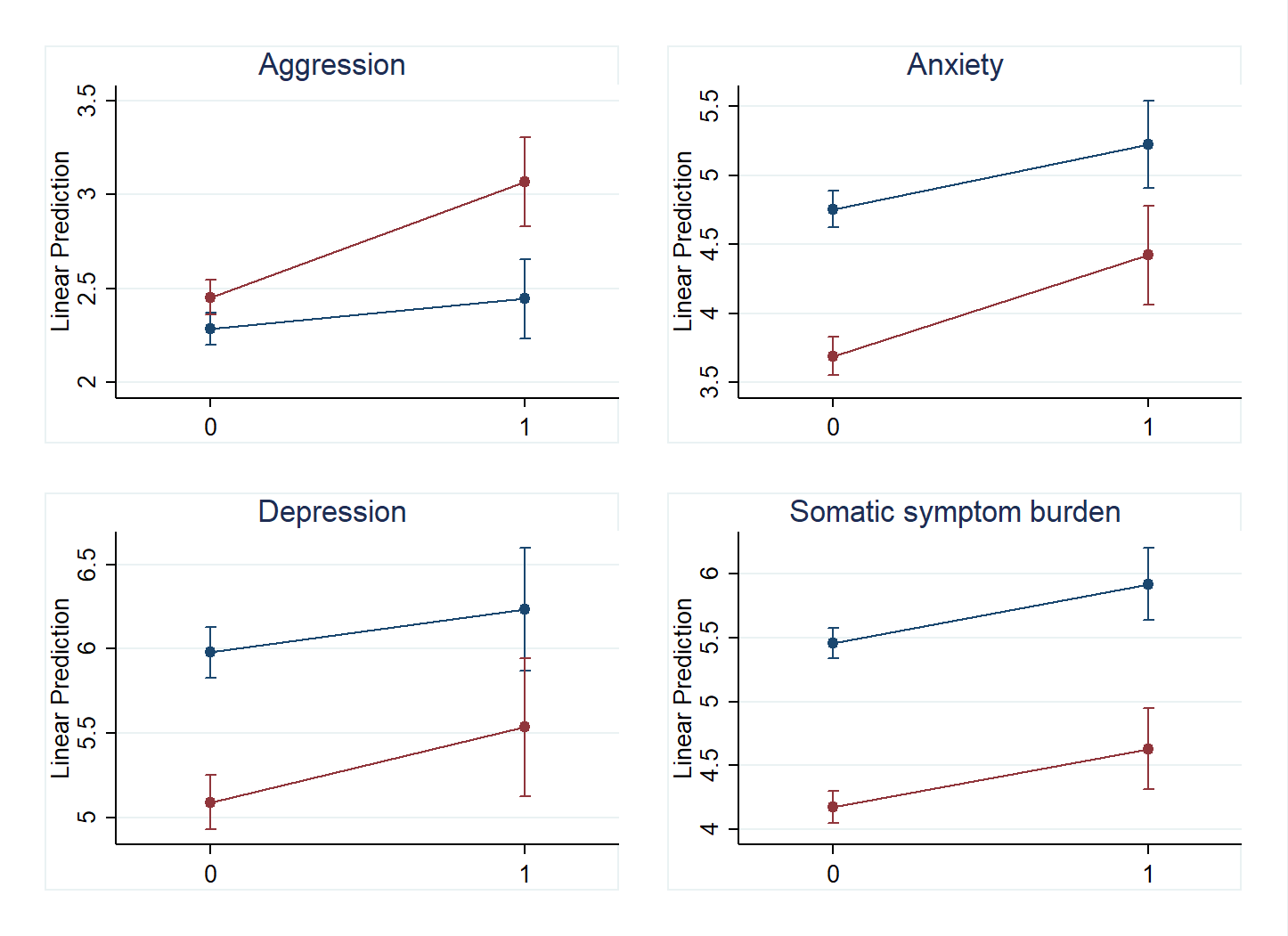


## GD in mental health and corona exposure (income decrease)

|  | (1) | (2) | (3) | (4) |
| --- | --- | --- | --- | --- |
| VARIABLES | Aggression | Anxiety | Depression | Somatic symptom burden |
|  |  |  |  |  |
| Gender: Male | 0.081 | -1.048*** | -0.932*** | -1.394*** |
|  | (0.068) | (0.101) | (0.117) | (0.093) |
| Income decreased | 0.719*** | 2.390*** | 2.548*** | 0.246* |
|  | (0.098) | (0.145) | (0.167) | (0.133) |
| Male x Income decreased | 0.518*** | -0.162 | 0.041 | 0.400** |
|  | (0.141) | (0.209) | (0.241) | (0.191) |
|  |  |  |  |  |
| Observations | 10,979 | 10,979 | 10,979 | 10,979 |
| R-squared | 0.045 | 0.086 | 0.083 | 0.034 |
| Model | Linear | Linear | Linear | Linear |
| F(1, 10977) | 74.42 | 147.1 | 141.9 | 55.20 |

Standard errors in parentheses

*** p<0.01, ** p<0.05, * p<0.1

*Controls*: age, household size, years of education, urban vs rural; data was weighted on age, gender and level of education.


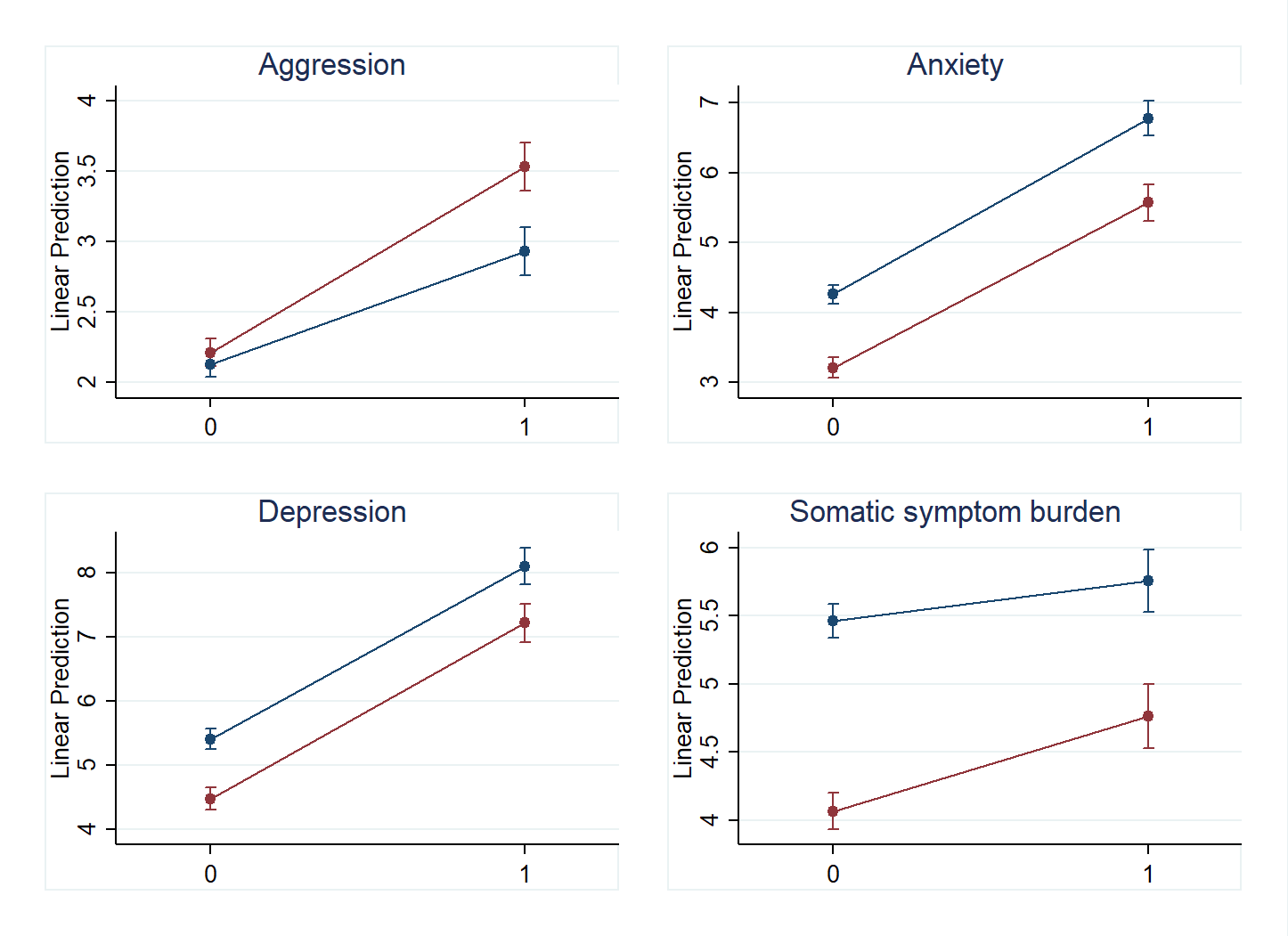


## GD in mental health and household characteristics (main provider)

|  | (1) | (2) | (3) | (4) |
| --- | --- | --- | --- | --- |
| VARIABLES | Aggression | Anxiety | Depression | Somatic symptom burden |
|  |  |  |  |  |
| Gender: Male | -1.056* | -0.653 | 0.979 | -1.255 |
|  | (0.593) | (0.890) | (1.020) | (0.805) |
| Main provider: me | -0.981* | -0.437 | -0.614 | 0.459 |
|  | (0.571) | (0.856) | (0.981) | (0.774) |
| Stringency | 0.015*** | 0.040*** | 0.051*** | 0.006 |
|  | (0.005) | (0.007) | (0.008) | (0.007) |
| Male x Main provider: me | 0.257 | 0.254 | -0.657 | -1.270 |
|  | (0.834) | (1.250) | (1.434) | (1.131) |
| Male x Stringency | 0.015* | -0.006 | -0.025* | 0.001 |
|  | (0.008) | (0.012) | (0.013) | (0.010) |
| Main provider: me x Stringency | 0.013* | 0.013 | 0.023* | -0.001 |
|  | (0.007) | (0.011) | (0.013) | (0.010) |
| Male x Main provider: me x Stringency | -0.001 | -0.004 | 0.005 | 0.013 |
|  | (0.011) | (0.016) | (0.018) | (0.015) |
|  |  |  |  |  |
| Observations | 10,979 | 10,979 | 10,979 | 10,979 |
| R-squared | 0.039 | 0.058 | 0.065 | 0.034 |
| Model | Linear | Linear | Linear | Linear |
| F(1, 10977) | 40.32 | 61.50 | 69.77 | 34.61 |

Standard errors in parentheses

*** p<0.01, ** p<0.05, * p<0.1

*Controls*: age, household size, years of education, urban vs rural; data was weighted on age, gender and level of education.


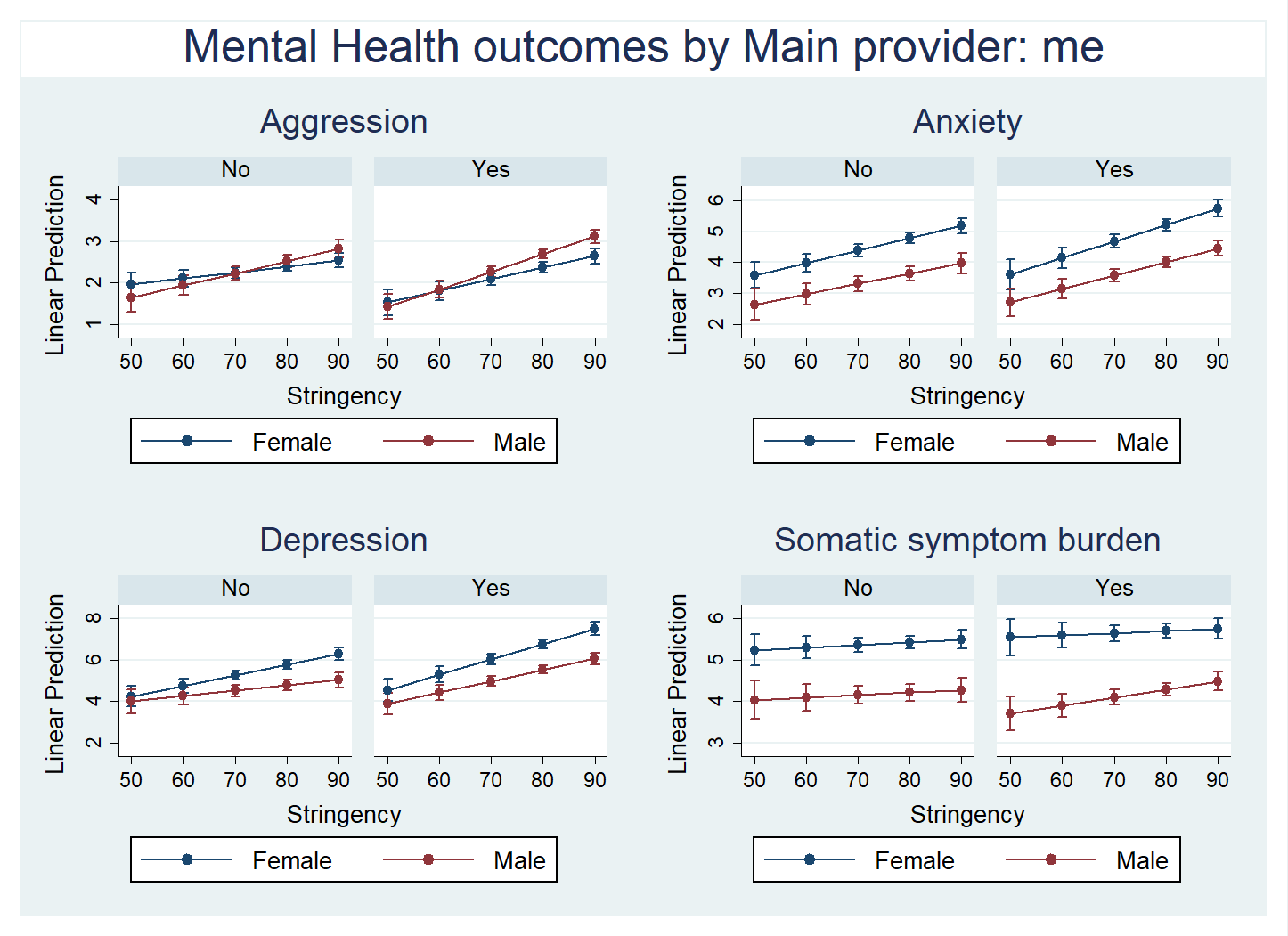


## GD in mental health and household characteristics (lives with children)

|  | (1) | (2) | (3) | (4) |
| --- | --- | --- | --- | --- |
| VARIABLES | Aggression | Anxiety | Depression | Somatic symptom burden |
|  |  |  |  |  |
| Gender: Male | -0.894* | 0.185 | 1.262 | -2.371*** |
|  | (0.469) | (0.705) | (0.810) | (0.636) |
| Lives with children | 1.311* | 1.570 | 0.948 | 0.002 |
|  | (0.674) | (1.013) | (1.164) | (0.914) |
| Stringency | 0.022*** | 0.052*** | 0.068*** | 0.007 |
|  | (0.004) | (0.006) | (0.007) | (0.006) |
| Male x Lives with children | -0.852 | -3.516** | -3.635** | 1.876 |
|  | (0.982) | (1.477) | (1.698) | (1.334) |
| Male x Stringency | 0.014** | -0.017* | -0.028*** | 0.015* |
|  | (0.006) | (0.009) | (0.010) | (0.008) |
| Lives with children x Stringency | -0.012 | -0.023* | -0.020 | -0.001 |
|  | (0.009) | (0.013) | (0.015) | (0.012) |
| Male x Lives with children x Stringency | 0.013 | 0.049*** | 0.049** | -0.030* |
|  | (0.013) | (0.019) | (0.022) | (0.017) |
|  |  |  |  |  |
| Observations | 10,979 | 10,979 | 10,979 | 10,979 |
| R-squared | 0.041 | 0.055 | 0.058 | 0.034 |
| Model | Linear | Linear | Linear | Linear |
| F(1, 10977) | 42.66 | 58.32 | 61.54 | 34.97 |

Standard errors in parentheses

*** p<0.01, ** p<0.05, * p<0.1

*Controls*: age, household size, years of education, urban vs rural; data was weighted on age, gender and level of education.


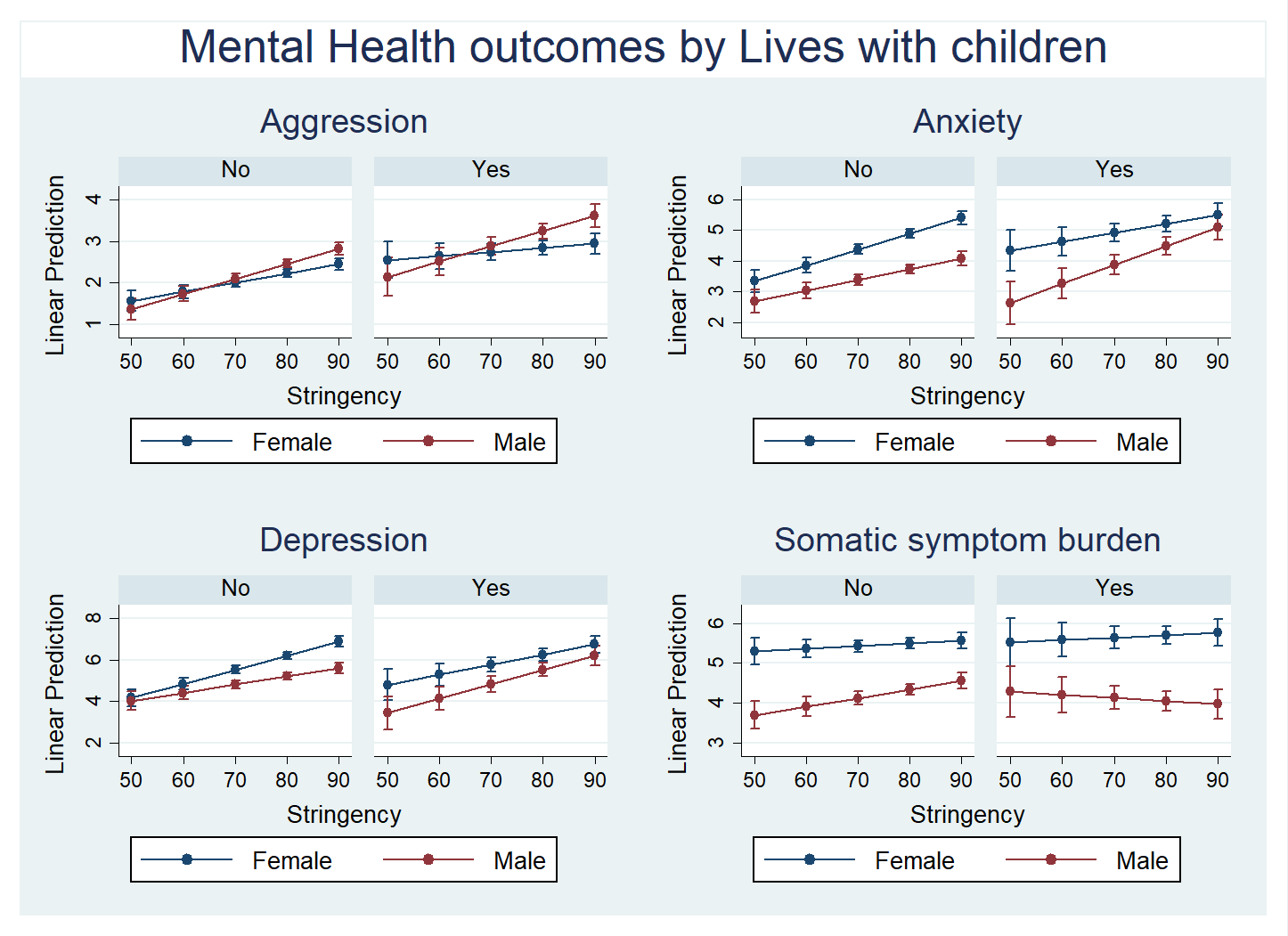


## GD in mental health and household characteristics (living alone)

|  | (1) | (2) | (3) | (4) |
| --- | --- | --- | --- | --- |
| VARIABLES | Aggression | Anxiety | Depression | Somatic symptom burden |
|  |  |  |  |  |
| Gender: Male | -1.285*** | -1.340* | -0.415 | -1.756*** |
|  | (0.477) | (0.714) | (0.816) | (0.646) |
| Lives alone | -1.374** | -0.951 | -0.607 | 0.075 |
|  | (0.642) | (0.963) | (1.100) | (0.870) |
| Stringency | 0.016*** | 0.041*** | 0.055*** | 0.005 |
|  | (0.004) | (0.006) | (0.007) | (0.006) |
| Male x Lives alone | 0.641 | 2.742* | 3.203** | -0.781 |
|  | (0.950) | (1.425) | (1.627) | (1.287) |
| Male x Stringency | 0.018*** | 0.002 | -0.008 | 0.005 |
|  | (0.006) | (0.009) | (0.010) | (0.008) |
| Lives alone x Stringency | 0.017** | 0.019 | 0.024* | 0.004 |
|  | (0.008) | (0.012) | (0.014) | (0.011) |
| Male x Lives alone x Stringency | -0.003 | -0.029 | -0.030 | 0.016 |
|  | (0.012) | (0.018) | (0.021) | (0.017) |
|  |  |  |  |  |
| Observations | 10,979 | 10,979 | 10,979 | 10,979 |
| R-squared | 0.039 | 0.059 | 0.074 | 0.036 |
| Model | Linear | Linear | Linear | Linear |
| F(1, 10977) | 40.61 | 62.94 | 79.39 | 37.48 |

Standard errors in parentheses

*** p<0.01, ** p<0.05, * p<0.1

*Controls*: age, household size, years of education, urban vs rural; data was weighted on age, gender and level of education.


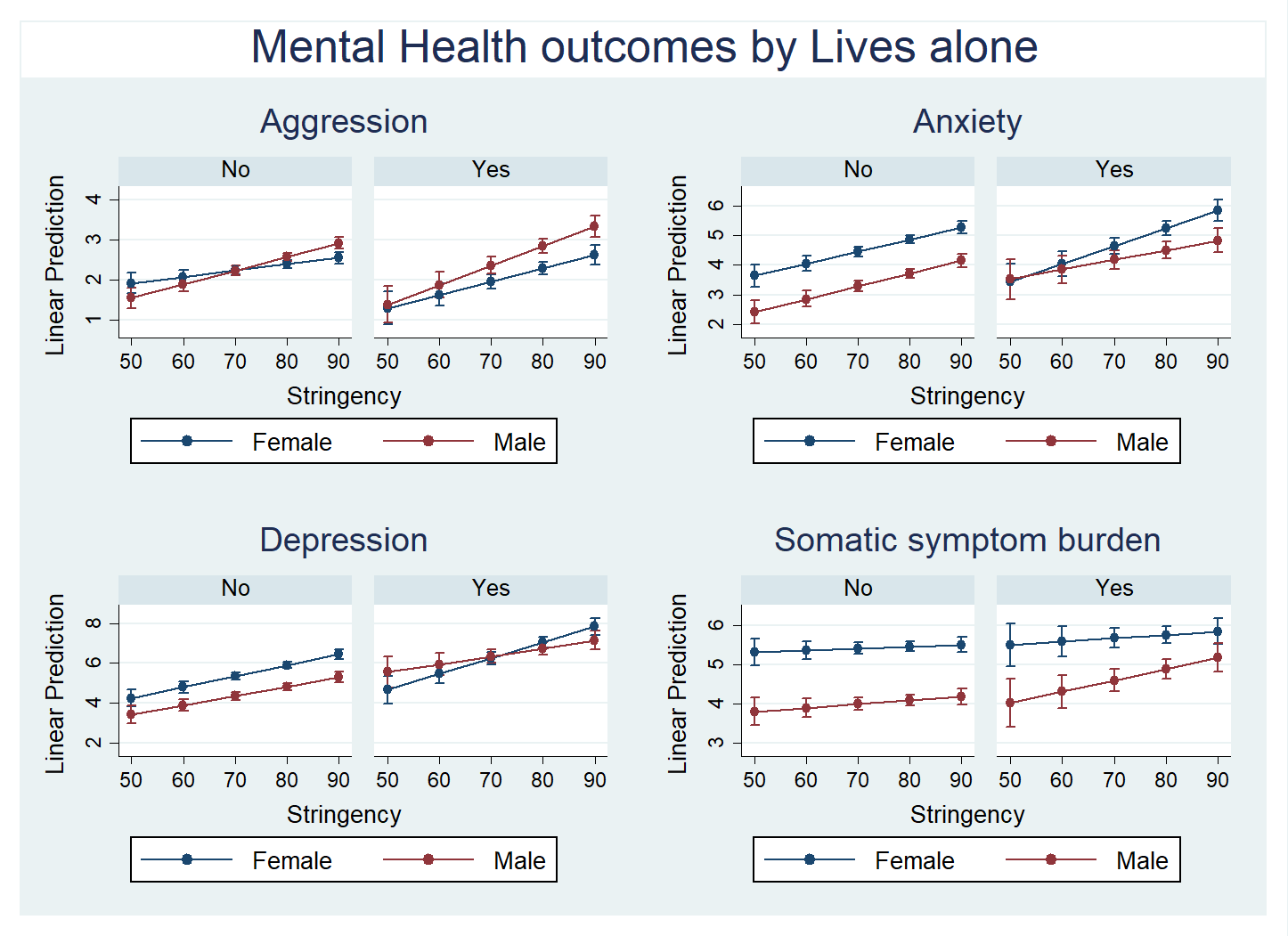

Supplement: Supplementary file 1 [file Data_Sheet_1.docx]
